# Enhanced Performance of Li–S Batteries via Dual Cathode–Interlayer Engineering: Hollow TiO_2_–Sulfur with Electrospun MXene–TMO Interlayers

**DOI:** 10.1021/acsomega.5c11112

**Published:** 2026-02-17

**Authors:** Busra Cetiner, Shungui Deng, Cesare Roncaglia, Thanya Phraewphiphat, Panpanat Tesatchabut, Adisak Promwicha, Daniele Passerone, Pimpa Limthongkul, Jakob Heier, Begum Yarar Kaplan, Selmiye Alkan Gursel, Alp Yurum

**Affiliations:** † Faculty of Engineering and Natural Sciences, Department of Materials Science and Nanoengineering, 52991 Sabanci University, 34956 Istanbul, Turkey; ‡ Laboratory for Functional Polymers, 28501 Empa, Swiss Federal Laboratories for Materials Science and Technology, 8600 Dubendorf, Switzerland; § Empa, Swiss Federal Laboratories for Materials Science and Technology, nanotech@surfaces Laboratory, Ueberlandstrasse 129, 8600 Duebendorf, Switzerland; ∥ National Energy Technology Center, 61191 National Science and Technology Development Agency, 12120 Khlong Luang, Thailand; ⊥ SUNUM Nanotechnology Research Centre, Sabanci University, 34956 Istanbul, Turkey

## Abstract

Lithium–sulfur (Li–S) batteries suffer from rapid capacity fading due to the polysulfide (LiPS) shuttle, sluggish redox kinetics, and the formation of insulating discharge products. Here, we report a dual-engineering strategy that integrates a hydrogen-treated hollow TiO_2_ (H–TiO_2_) sulfur host with conductive poly­(vinylidene fluoride) (PVDF)-based MXene–TMO interlayers. Hydrogen treatment introduces Ti^3+^/oxygen vacancies and forms a hollow framework, imparting enhanced conductivity to TiO_2_ while providing abundant active sites for sulfur immobilization and redox catalysis. Complementarily, the best-performing MXene–TMO interlayer, PVDF/MXene–SnO_2_ (PV–MS), couples the high conductivity of MXene with the polar, catalytic activity of SnO_2_, enabling efficient LiPS adsorption and accelerated conversion. This synergy yields substantial performance improvements: LiPS charge-transfer resistance decreases by 93% (4.5 to 0.31 Ω), cycling stability is significantly enhanced (capacity retention >81% compared with 64% for the reference cell), Li^+^ diffusion rates nearly double, and fast kinetic reactions are maintained even at high scan rates without diffusion limitations. Additionally, the rate capability remains robust at high current densities. Density functional theory (DFT) calculations further confirm this synergistic behavior, showing that the adsorption free energy of Li_2_S_6_ follows the trend |Δ*G*_ads_|_H–TiO_2__ > |Δ*G*_ads_|_TiO_2__ > |Δ*G*_ads_|_graphene_, indicating the strongest LiPS binding and the highest catalytic reactivity on H–TiO_2_ surfaces. Both DFT and XPS analyses reveal a distinct dual-site binding mechanism in H–TiO_2_, where Ti–S and Ti–O–Li interactions cooperatively enhance polysulfide anchoring, promote faster redox conversion, and improve sulfur utilization. To the best of our knowledge, this is the first demonstration of a dual-engineered Li–S cathode system in which defect-mediated sulfur hosts and catalytic interlayers operate synergistically. The resulting mechanism—controlled sulfur release at the cathode, shuttle suppression at the interlayer, and rapid electron/ion transport across the interface, establishes a powerful design guideline for achieving long-lived and high-rate Li–S batteries.

## Graphical abstract

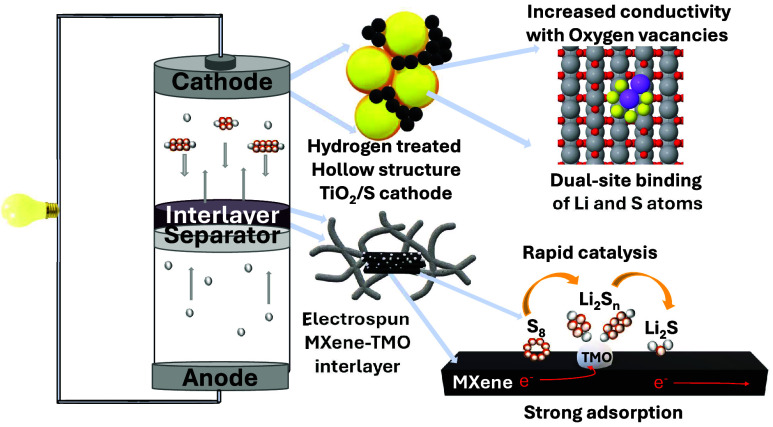

## Introduction

Lithium–sulfur (Li–S) batteries have emerged as a highly promising energy storage technology due to their earth-abundant and low-cost materials, environmental compatibility, and remarkable theoretical metrics, including a specific capacity of 1675 mAh/g and a specific energy of 2600 Wh/kg.^1^ Despite this potential, Li–S batteries face several intrinsic limitations that hinder their practical implementation. The low electrical conductivity of sulfur, combined with its large volumetric expansion during cycling, leads to sluggish electrochemical kinetics and rapid capacity fading. In addition, incomplete sulfur utilization further restricts the achievable specific capacity.

Among these challenges, the migration of lithium polysulfides (LiPSs) from the cathode to the anode—commonly referred to as the shuttle effect—remains one of the most critical issues undermining long-term cycling stability and Coulombic efficiency.^2^ The dissolution of LiPS intermediates disrupts the uniform deposition of reaction products, causing sulfur agglomeration and blockage of the electrode/electrolyte interface. These effects contribute to large overpotentials during the solid–liquid–solid conversion process.^3^

To address the shuttle effect in Li–S batteries, efforts have increasingly focused on confining LiPSs through physical, chemical, or deliberately coupled mechanisms. Physical routes leverage carbonaceous hosts and porous interlayers to impede LiPS diffusion through nanoscale pores and extended diffusion pathways.^4,5^ Chemical routes employ polar and catalytic hosts—particularly metal oxides such as TiO_2_, SnO_2_, and Co_7_Fe_3_—to immobilize LiPSs through strong interactions while accelerating their redox conversion. Building on these foundations, functional interlayers integrate architectural control with targeted chemistry, yielding more persistent LiPS retention and improved reaction kinetics.

Carbon-based hosts, including carbon nanotubes,^6^ mesoporous carbons,^7^ carbon fibers,^8^ and graphene derivatives,^9,10^ have been widely investigated for encapsulating sulfur into carbon matrices to achieve physical confinement. These sulfur/carbon composites provide effective physical confinement of sulfur and LiPSs through porous architectures.

Nevertheless, during prolonged cycling, hydrophilic LiPS species tend to diffuse out of the hydrophobic carbon framework. This limitation arises from the intrinsically nonpolar nature of carbon, which interacts only weakly with polar polysulfides.^11^ To overcome this drawback, more targeted approaches exploit chemical interactions between LiPSs and oxygen-containing functional groups at the host interface.

To overcome the weak affinity of nonpolar carbons toward LiPSs, polar hosts and catalytic materials have been widely explored, as LiPSs are intrinsically polar species with terminal sulfur atoms carrying most of the negative charge. Metal oxides, in particular, offer abundant polar sites that interact strongly with LiPSs through polar–polar interactions, Lewis acid–base chemistry, and sulfur-chain catenation, thereby immobilizing them within or on the host surface.^11^ Representative transition-metal oxides (TMOs), such as TiO_2_,^12^ Ti_4_O_7_,^11^ MnO_2_,^13^ NiFe_2_O_4_,^14^ SnO_2_,^15^ MgO,^16^ ZnS,^17^ and NiO,^18^ have thus attracted significant attention as sulfur hosts. Among these oxides, TiO_2_ is especially attractive for its abundance, stability, and ability to provide both physical confinement and chemical anchoring of LiPSs.^19^ However, its intrinsically low conductivity limits electrochemical performance, prompting defect engineering approaches such as hydrogen treatment to enhance the conductivity and strengthen LiPS adsorption.

Hydrogen-treated hollow TiO_2_ (H-TiO_2_) builds upon this strategy by simultaneously enhancing the electronic conductivity and reinforcing chemical interactions with LiPSs. The introduction of Ti^3+^ sites and oxygen vacancies during hydrogen reduction not only accelerates electron and lithium-ion transport but also promotes strong adsorption and catalytic conversion of polysulfides.^20^ Coupled with the 3D hollow framework and thin TiO_2_ shell, H-TiO_2_ effectively integrates physical confinement with chemical anchoring, suppressing shuttle effects, accommodating volume changes, and delivering improved redox kinetics and long-term cycling stability.^21^ Collectively, H-TiO_2_ emerges as a highly promising sulfur host, where enhanced electronic conductivity, strengthened LiPS adsorption, and confined cavities for controlled sulfur loading and release—similar to drug-delivery systems, synergistically provide a robust platform for high-performance Li–S cathodes.

Another effective strategy to mitigate the shuttle effect is the introduction of functional interlayers, which act as selective barriers between the cathode and the separator to confine LiPSs and provide additional space for their redistribution during cycling.^2^ Such interlayers not only suppress polysulfide migration but also reduce cell resistance and improve sulfur utilization by combining physical restriction with chemical anchoring. Carbon-based interlayers—including graphene, carbon nanotubes,^22^ and carbon papers^23^—have been extensively investigated; however, their nonpolar nature results in weak interactions with polar LiPSs, leading to incomplete suppression. TMOs such as TiO_2_,^24^ SnO_2_,^25^ MnO_2_,^26^ MoS_2_,^27^ ZrO_2_,^28^ and V_2_O_5_ ^29^ offer stronger chemical affinity but are often limited by their relatively small surface area and poor conductivity. To overcome these shortcomings, MXenes have recently emerged as promising candidates owing to their two-dimensional layered structure, large surface area, high conductivity, and abundant active sites.^30^ MXene-based interlayers accelerate ion transport, facilitate electron transfer, and lower the energy barrier for LiPS conversion, although their integration with TMOs for synergistic effects has remained largely unexplored.^31,32^

Electrospinning provides an attractive pathway to engineer such multifunctional interlayers, enabling the fabrication of free-standing fibrous membranes with interconnected porosity, tunable fiber diameter, and high mechanical flexibility.^33^ The porous networks promote electrolyte infiltration and fast ion diffusion while physically confining LiPSs, thereby reducing shuttle-induced losses. In addition, incorporating MXene–TMO heterostructures into electrospun membranes offers the dual benefits of strong polysulfide chemisorption and rapid charge transport.^34,35^ Within this framework, poly­(vinylidene fluoride) (PVDF) serves as a robust polymer matrix, providing polar functional groups for enhanced polysulfide trapping alongside chemical stability and mechanical strength to maintain structural integrity during long-term cycling.

Here, we propose a dual-engineering strategy for Li–S cathodes that, to the best of our knowledge, is reported for the first time. By integrating H-TiO_2_ hosts with electrospun MXene–TMO interlayers, our approach introduces a drug-delivery-inspired sulfur-release mechanism alongside a highly conductive, polysulfide-trapping barrier. The hollow TiO_2_ framework not only regulates sulfur utilization through gradual release but also accelerates redox kinetics via abundant oxygen vacancies. Meanwhile, the MXene–TMO interlayer acts as a multifunctional shield, synergistically combining physical confinement, chemical anchoring, and rapid electron/ion transport. This dual platform delivers a powerful solution to the long-standing shuttle effect, simultaneously boosting conductivity, stabilizing active material, and enabling superior cycling stability—paving the way toward practical high-energy Li–S batteries.

## Results and Discussion

### Morphology and Structural Characterization

Electrospun fiber-based interlayers were successfully fabricated from PVDF–MXene (PV-MX), PVDF–Calcined MXene (PV-CM), and PVDF–MXene/SnO_2_ (PV-MS) hybrids. The characterization studies of CM are listed in Figure S1. As shown in Figure 1a–c, the electrospinning process yielded continuous, bead-free fibers with a highly porous and interconnected network, providing efficient ionic transport channels and tunable pore structures. Notably, the incorporation of MXene and MXene–SnO_2_ nanoparticles occurred in a dual manner: they were homogeneously distributed across the fiber surfaces while also being partially embedded within the fiber interiors (Figure 2). Such a fiber–MXene architecture is consistent with previous reports.^36^

**Figure 1:**
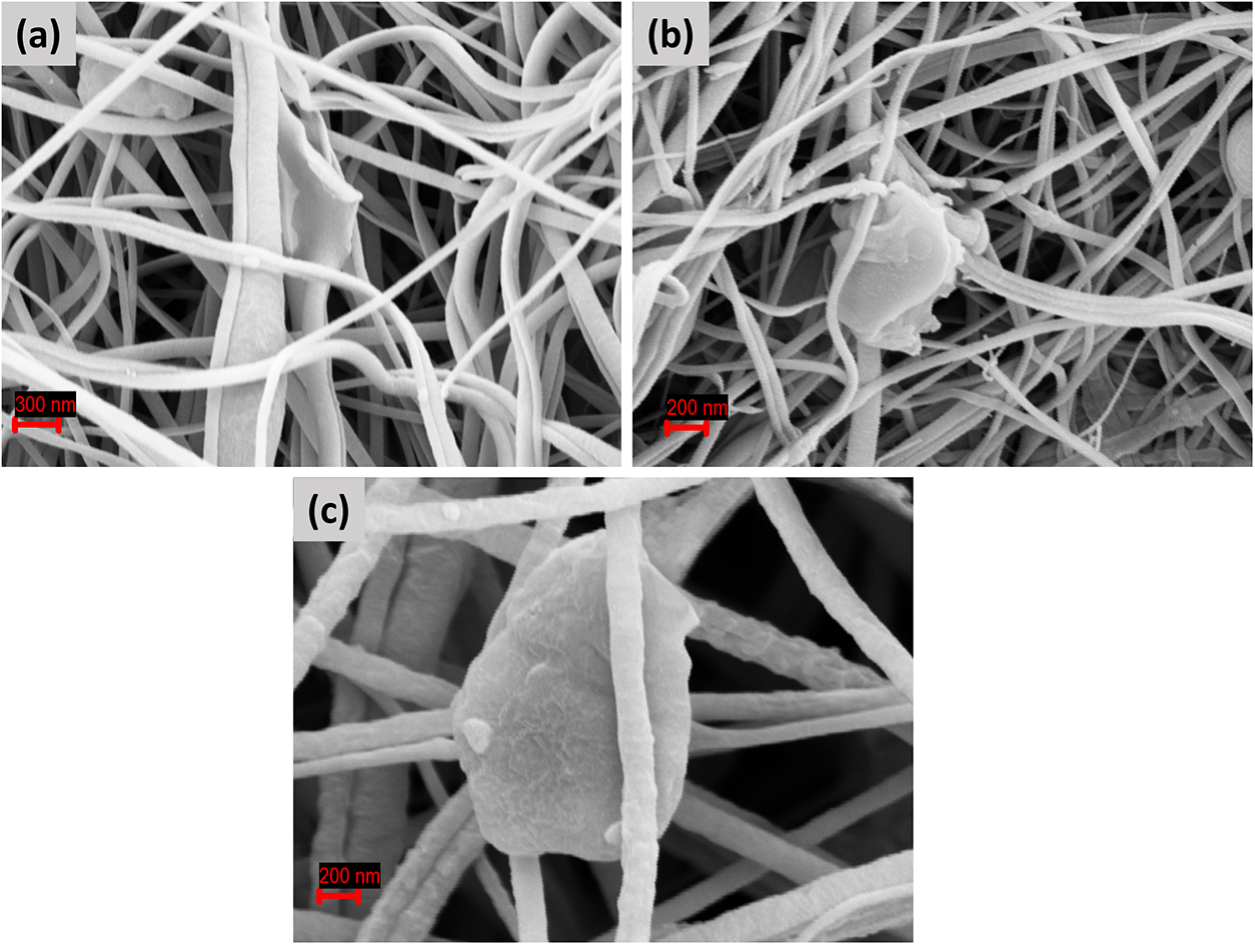
SEM (secondary electron, SE) analysis of electrospun PVDF-based interlayers: (a) PV-MX, (b) PV-CM, and (c) PV-MS. The SE images show the fibrous morphology and uniform dispersion of the MXene-TMO structures within the PVDF matrix.

**Figure 2:**
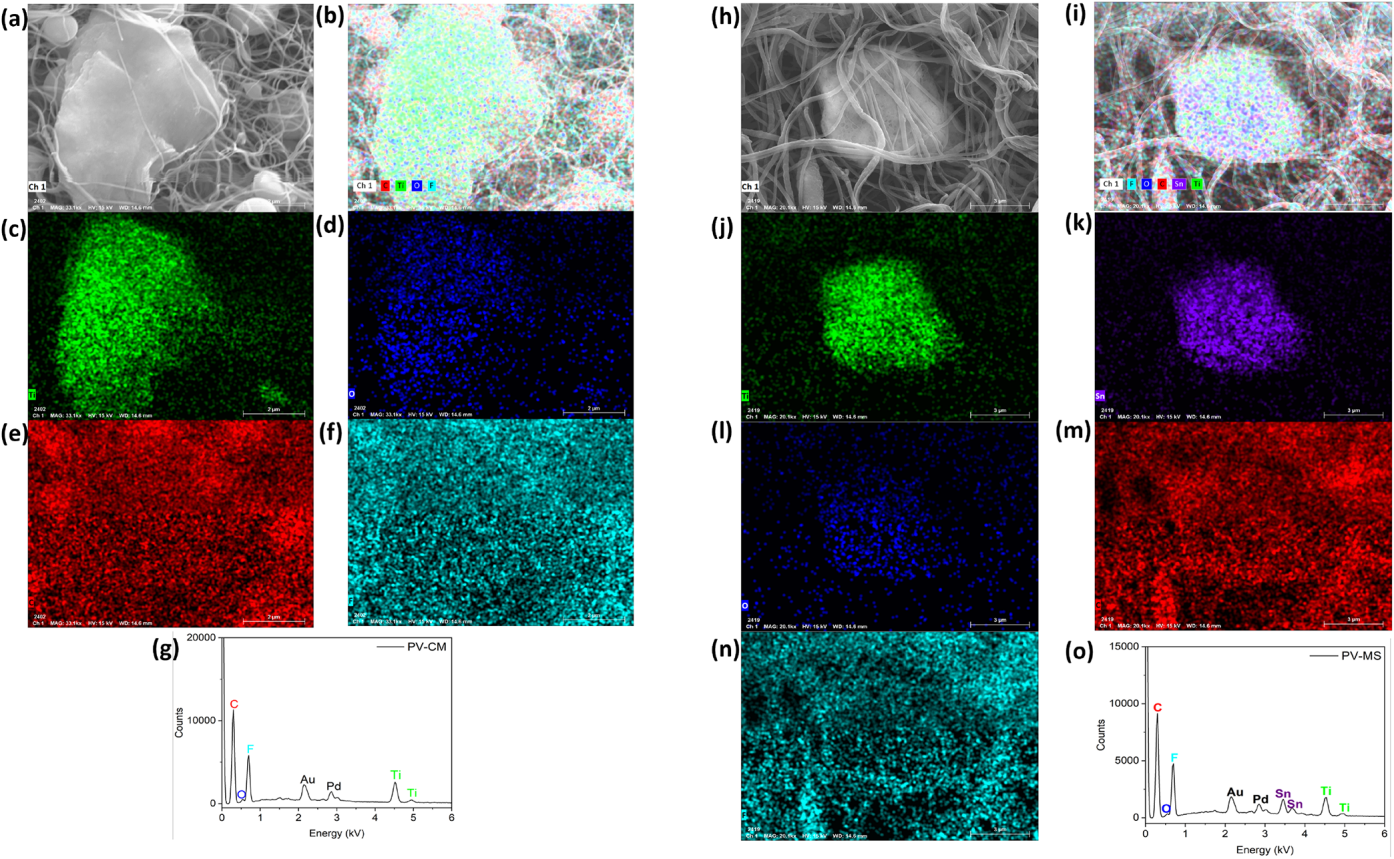
SEM and EDS elemental mappings of electrospun PVDF-based interlayers. PV-CM sample: (a, b) SEM image, (c–f) elemental distribution maps of Ti, O, C, and F; (g) corresponding EDS spectrum. PV-MS sample: (h, i) SEM images; (j–o) elemental distribution of Ti, Sn, O, C, and F; (p) corresponding EDS spectrum.

This hierarchical integration ensures strong physical confinement of LiPSs, while the polar nature of PVDF and the catalytic MXene-based domains synergistically promote their adsorption and accelerated redox conversion. The calcined MXene further provides defect-rich, high-surface-energy sites that intensify chemical anchoring, whereas SnO_2_ nanoparticles introduce additional polar–polar interactions and catalytic activity.^37^ Moreover, EDS elemental mapping reveals that the MXene particles contain surface oxygen. It is well established that O-functionalized MXene surfaces can facilitate the conversion of liquid LiPSs (Li_2_S_*x*_, *x* > 3) into insoluble sulfur species.^38^

Collectively, this multifunctional interlayer design not only mitigates polysulfide shuttling but also enhances the reaction kinetics, thereby enabling long-term stability and high-rate capability in Li–S batteries.

SEM analysis of the H-TiO_2_ spheres before cycling, shown in Figure 3, reveals predominantly well-defined hollow architectures (average particle diameters of 320–350 nm), which are clearly visible in partially fractured particles. The hollow interiors and hierarchical shells of the spheres indicate the effectiveness of the templating method used for TiO_2_ synthesis and suggest a controlled structural evolution. Although some unconverted domains remain, most spheres display a porous, interconnected nanocrystalline framework that provides abundant channels for electrolyte infiltration and LiPS adsorption.

**Figure 3:**
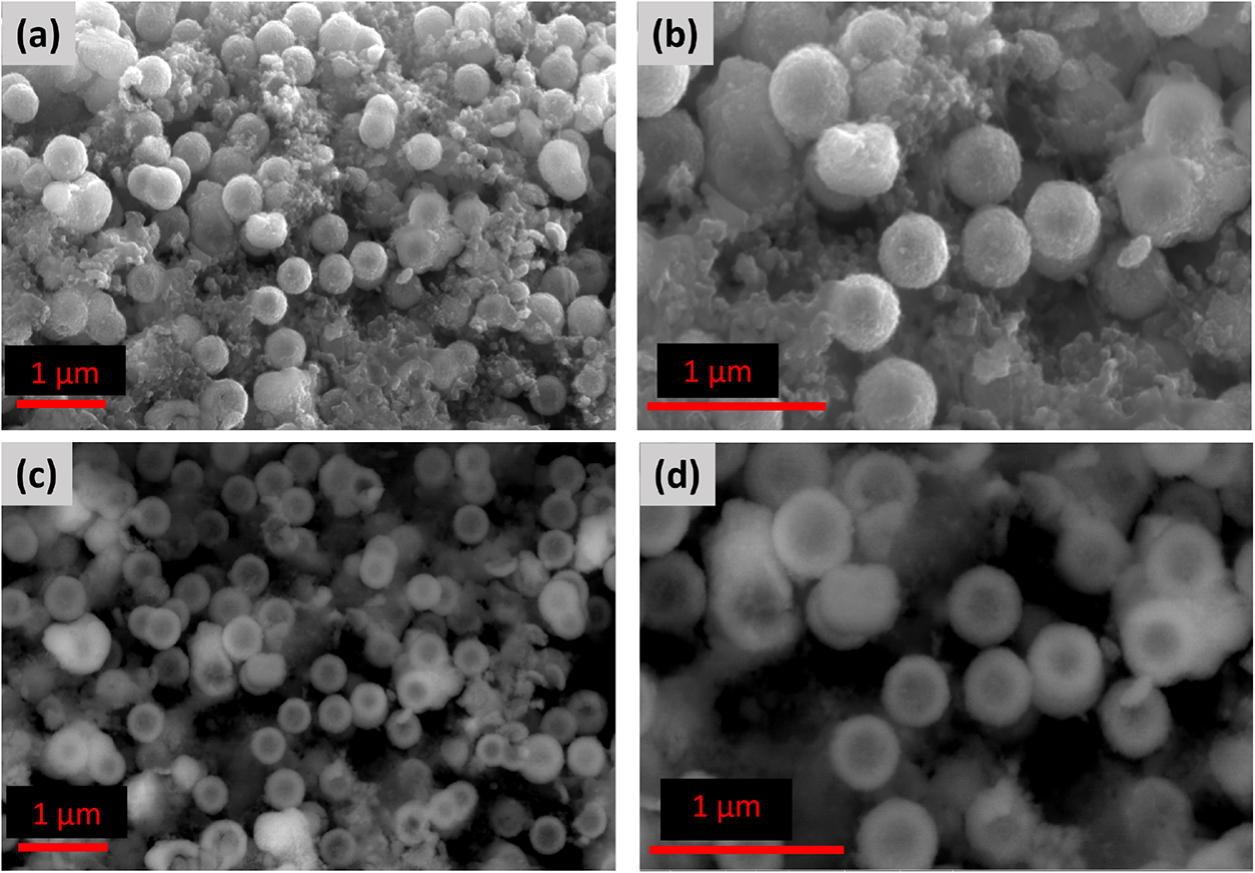
SEM characterization of the H–TiO_2_ structure before cycling. (a, b) Secondary electron (SE) images showing the surface morphology of the H-TiO_2_ spheres at different magnifications. (c, d) Backscattered electron (BSE) images highlighting the shell density contrast and the hollow interior structure.

Notably, small carbon particles—intentionally employed as conductive binders—are observed surrounding the TiO_2_ spheres, whereas only negligible amounts of sulfur residues appear externally. This hollow, hierarchically porous configuration is expected not only to enhance polysulfide confinement but also to maximize interfacial contact for subsequent redox processes, thereby offering a significant advantage over conventional TiO_2_ morphologies.

After cycling, the H–TiO_2_ electrode exhibits notable structural evolution (Figure 4). In addition to retaining the overall spherical framework, thin web-like deposits are observed on the surface, which are attributed to solid Li_2_S species formed during cycling. These deposits partially cover the surface, indicating the active involvement of the hollow spheres in polysulfide conversion reactions. Importantly, the TiO_2_ spheres largely preserve their morphology, with fractured particles still revealing the underlying hollow interior.

**Figure 4:**
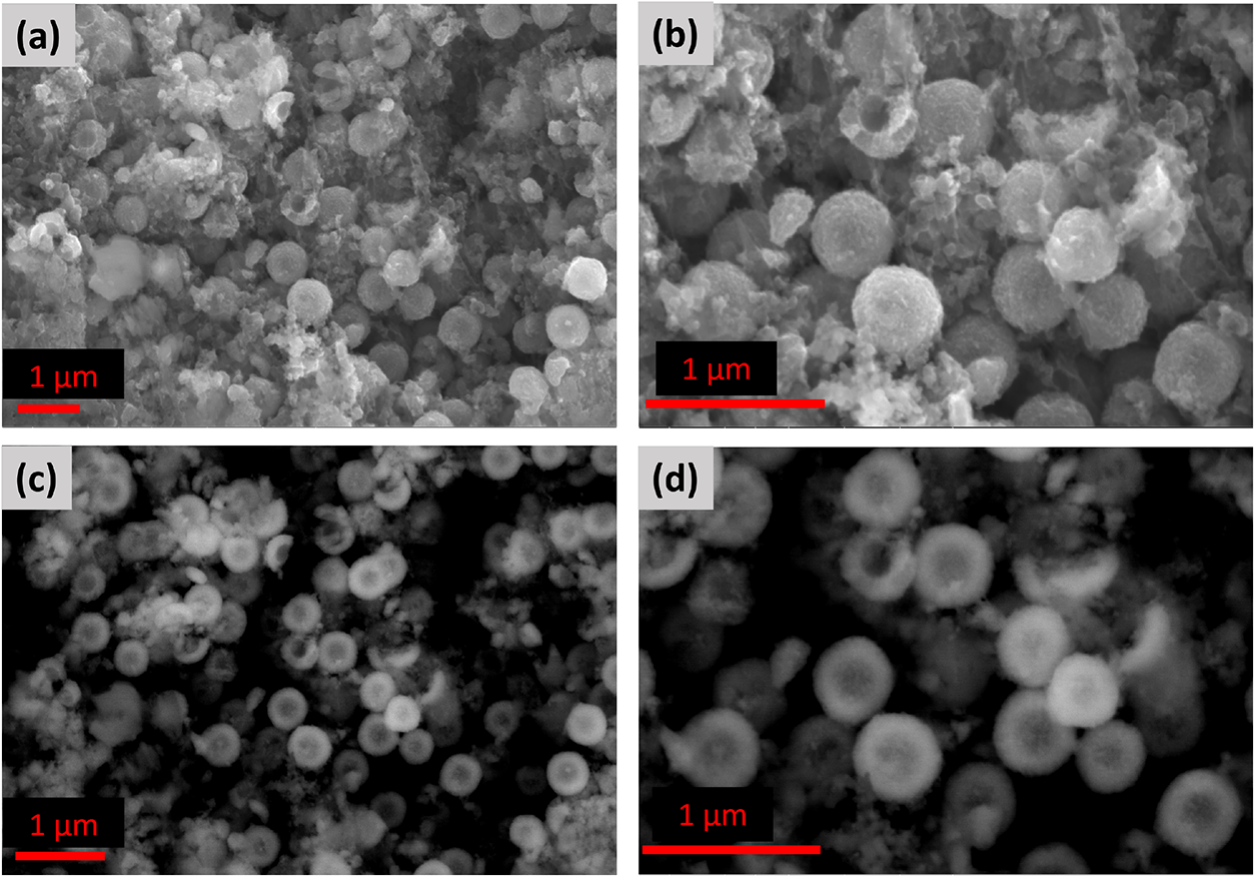
SEM characterization of the H–TiO_2_ structure after 50 cycles. (a, b) Secondary electron (SE) images illustrating the cycled H–TiO_2_ surface morphology and structural integrity at different magnifications. (c, d) Backscattered electron (BSE) images highlighting compositional contrast and the preserved hollow architecture after extended electrochemical cycling.

A moderate increase in particle size is observed—from 320 to 350 nm before cycling to 390–420 nm afterward. This increase reflects the volume expansion typically associated with Li–S conversion chemistry; however, the relatively small expansion in our system is attributed to the hollow and porous architecture of the TiO_2_ spheres. The internal void space accommodates the formation of Li_2_S and mitigates excessive structural strain. The observed growth may also stem from surface reactions and Li_2_S deposition.

Despite these changes, the porous architecture remains clearly visible, demonstrating that the hollow and porous characteristics of H–TiO_2_ are effectively maintained after prolonged cycling, thereby preserving continuous ion/electron transport pathways and structural integrity.

The XRD patterns of the H–TiO_2_ cathodes before and after cycling are presented in Figure 5. Prior to cycling, the cathode exhibits the characteristic reflections of rutile TiO_2_, with diffraction peaks indexed to the (110), (101), (111), (210), (211), and (220) planes at 2θ = 29.4, 36.1, 43.4, 47.4, 56.7, and 57.6°, respectively.^39^

**Figure 5:**
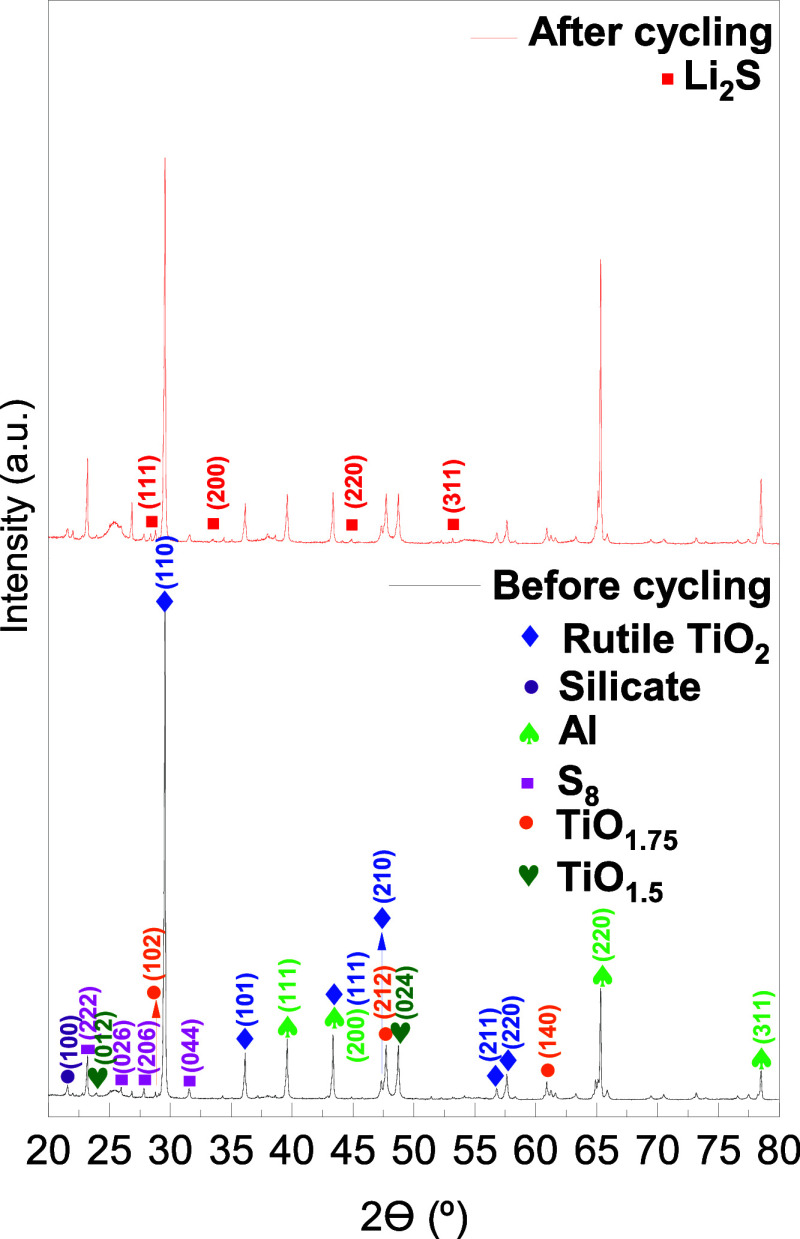
XRD patterns of the H–TiO_2_/S cathodes before and after cycling, compared with standard reference patterns of rutile TiO_2_/S (JCPDS No. 79-5860), orthorhombic α-sulfur S_8_ (JCPDS No. 78-1889), and Li_2_S (PDF No. 23-0369). The confinement-induced peak shifts and the appearance of low-intensity α-S_8_ reflections are highlighted for the sake of clarity.

The characteristic diffraction peaks of orthorhombic α-sulfur (S_8_) appear at 23.2, 25.9, 27.9, and 31.6°, indexed to the (222), (026), (206), and (044) planes, respectively.^40^ In our sample, these weak reflections exhibit a slight shift toward lower 2θ values relative to the standard JCPDS PDF No. 78-1889, a well-established effect associated with lattice distortion and confinement when sulfur is embedded within nanoscale or hollow polar hosts.^41^ Most sulfur remains XRD-amorphous due to nanoscale dispersion within TiO_2_, consistent with previously reported TiO_2_/S composite systems.

The rutile reflections of H-TiO_2_ exhibit a systematic shift toward higher 2θ values compared to the standard rutile reference (JCPDS No. 88-1172), whereas the silica peak remains unchanged. This selective right-shift reflects a slight lattice contraction caused by hydrogen-induced Ti^3+^ formation, oxygen vacancies, and curvature-driven compressive strain intrinsic to hollow shell geometries. Hydrogen treatment reduces Ti–O bond lengths, and the concave geometry of the shells enhances compressive strain during cooling.^42,43^ The presence of substoichiometric reduced titanium oxides is also confirmed by weak reflections associated with Ti_4_O_7_ (TiO_1.75_) at 28.8, 47.7, and 60.9° ^11,44^ and Ti_2_O_3_ (TiO_1.5_) at 23.9 and 48.7°.^45^ These findings are fully consistent with the Ti^3+^ signatures observed in XPS. Additionally, reflections from the aluminum current collector appear at 39.6, 43.3, 65.2, and 78.5°, indexed to the (111), (200), (220), and (311) planes of Al,^46^ confirming their expected presence in the electrode stack.

In addition, very weak reflections originating from residual silica are observed at 21.5° indexed to the (100) plane of silica-based species.^47,48^ This signal stems from the Stöber SiO_2_ hard template used in forming the hollow TiO_2_ spheres. As widely reported for template-derived hollow TiO_2_ structures, trace silicate residues often persist after alkaline etching; however, they are electrochemically inert and do not participate in polysulfide adsorption or redox reactions.

Overall, the combined XRD signals confirm the coexistence of rutile TiO_2_, reduced TiO_*x*_ phases, confined sulfur, trace silica residues, and Al substrate contributions.

After cycling, new diffraction peaks appear at 28.4, 33.5, 44.0, and 53.2°, corresponding to the (111), (200), (220), and (311) planes of crystalline Li_2_S.^49^ The emergence of Li_2_S confirms the electrochemical conversion of sulfur species during discharge, while the persistence of rutile reflections demonstrates the structural stability of the H–TiO_2_ host throughout cycling.

The XPS spectra provide crucial insights into the surface chemistry and depth-dependent evolution of hollow-structured H–TiO_2_/S cathodes before and after cycling, as shown in Figure 6. For the pristine sulfur states, the spectra reveal distinct contributions from elemental sulfur, with S_B_ assigned to S_8_ formation at 163.7 and 164.9 eV, and S_T_ corresponding to S–Ti–S bonds at 161.7 and 162.9 eV.^50^ In H–TiO_2_, the presence of Ti^4+^, Ti^3+^, and even Ti^2+^ species—arising from the hydrogen-induced reduction of Ti^4+^—is clearly identified.

**Figure 6:**
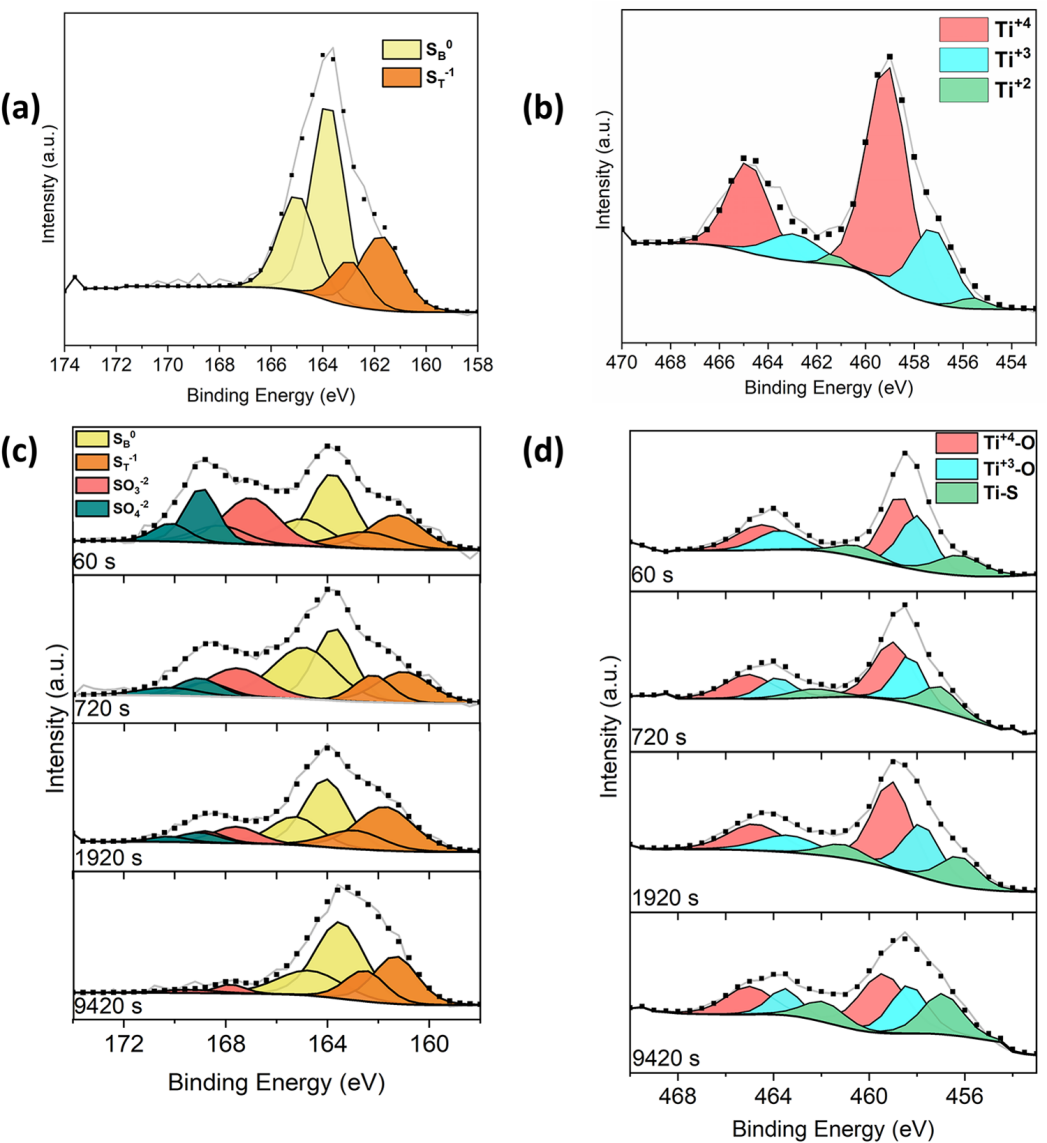
High-resolution XPS spectra of H–TiO_2_ electrodes before and after cycling. (a, c) S 2p spectra obtained before (a) and after (c) cycling, showing the evolution of sulfur chemical states. (b, d) Ti 2p spectra recorded before (b) and after (d) cycling, illustrating changes in Ti^+^/Ti+3 surface states and oxygen-vacancy formation.

After cycling, the sulfur chemistry becomes more complex. In addition to the persistence of S–B (Li_2_S_*x*_, 2 < *x* ≤ 8) and S–T (Li_2_S_*x*_, *x* ≤ 2) bonds, additional peaks corresponding to lithium polysulfide residues and sulfate-like species emerge, confirming the formation of a cathode–electrolyte interphase (CEI). The presence of S–B species is consistent with previously reported XANES observations. Although our cell was analyzed in the charged state, Miller et al. reported a pronounced increase in polysulfide-related features at the beginning of the charging process following discharge, with a characteristic signal at approximately 2470.35 eV.^51^ At the end of the charging state, the intensity of these polysulfide peaks reached its maximum. In the charged state, the most dominant S–B species were identified as Li_2_S_8_ and a mixture of Li_2_S_6_ and Li_2_S_4_.

Additionally, all spectra exhibit a broad peak at 166.5 eV, attributed to SO_3_^–2^ species, and a more pronounced peak at 169.5 eV corresponding to SO_4_^–2^ groups, both arising from the decomposition of the TFSI anion.^52^ Importantly, depth-profile analysis reveals a pronounced sputtering effect: as the sputtering time increases, the intensity of salt-related species gradually decreases, confirming their localization primarily in the outer CEI layer. In parallel, the relative proportion of S-T species increases in deeper regions, reflecting the presence of unreacted sulfur within the hollow cavities. This depth-dependent sulfur redistribution underscores the critical role of the porous hollow framework in hosting and retaining active sulfur.

In the case of H-TiO_2_, the Ti 2p spectra shift to higher binding energies compared to pristine rutile TiO_2_, indicating the partial reduction of Ti^4+^ to Ti^3+^, the formation of oxygen vacancies,^11,53^ and the concurrent development of Ti–S bonds. Depth profiling further confirms this chemical evolution: after 60 s of sputtering, the surface composition consists of 46.34% Ti^4+^–O bonding at 458.49 eV, 35.03% Ti^3+^–O bonding at 457.9 eV, and 18.63% Ti–S bonding at 456.06 eV. With prolonged sputtering (9420 s), the relative contributions of Ti^4+^–O and Ti^3+^–O decrease slightly to 44.75 and 28.82%, respectively, while the Ti–S signal increases significantly to 26.43%.

This progressive enrichment of Ti–S species in the subsurface indicates that H-TiO_2_ actively adsorbs and immobilizes lithium polysulfides through strong Ti–S interactions. The decrease in Ti^3+^ content with depth suggests gradual filling or consumption of oxygen vacancies, likely due to their participation in polysulfide adsorption and redox reactions. The formation of Ti–S bonds is also supported by XRD, where the (110) rutile TiO_2_ peak shifts to lower diffraction angles. Moreover, hydrogen treatment enhances the diffusion of reactants within the hollow porous structure, leading to an increase in pore size^53^ and thereby improving the accessibility of active sites for polysulfide conversion.

Theoretically, a stronger adsorption capability in a host material corresponds to higher binding energies with lithium polysulfides (LiPSs). The introduction of oxygen vacancies and Ti^3+^ species into TiO_2_ enhances its electrical conductivity and facilitates faster ion and electron transport during LiPS redox conversion. Therefore, an ideal sulfur host should combine strong LiPS binding—ensuring effective immobilization, with a low kinetic barrier to promote subsequent conversion reactions. Such a balance is essential for suppressing the shuttle effect and overcoming the sluggish reaction kinetics that typically limit the performance of Li–S batteries.

In order to rationalize the beneficial effect of H-TiO_2_ in the sulfur cathode, the binding energy of the Li_2_S_6_ polysulfide on three different surfaces was calculated by using density functional theory (DFT). The details of the DFT setup and computational parameters are provided in the Methods section. The three surfaces selected for comparison were: carbon (graphene), the (110) surface of rutile TiO_2_, and the (110) surface of rutile TiO_2_ containing oxygen vacancies. As shown in Figure 7a,b, Li_2_S_6_ interacts only weakly with graphene, and its molecular structure remains almost unchanged from that of the gas phase. In contrast, its interaction with TiO_2_ is significantly stronger (Figure 7c,d), as indicated by molecular deformation and the corresponding adsorption free energies. The binding configuration and adsorption sites on H-TiO_2_ are presented in Figure 7e,f. These results clearly demonstrate that in both TiO_2_-based cases, not only the lithium atoms but also the sulfur atoms participate in binding to the substrate—unlike in graphene, where the interaction occurs primarily through Li atoms. This reveals a distinct and more effective dual-site binding mechanism involving strong Ti–S and Ti–O–Li interactions, which is further supported by XPS analysis (Figure 6d). Such interactions enhance polysulfide retention and immobilization, effectively trapping both solid sulfur species and lithium polysulfides, thereby suppressing the shuttle effect. Furthermore, they facilitate the redox conversion of polysulfides, accelerating reaction kinetics and improving sulfur utilization.^54^

**Figure 7:**
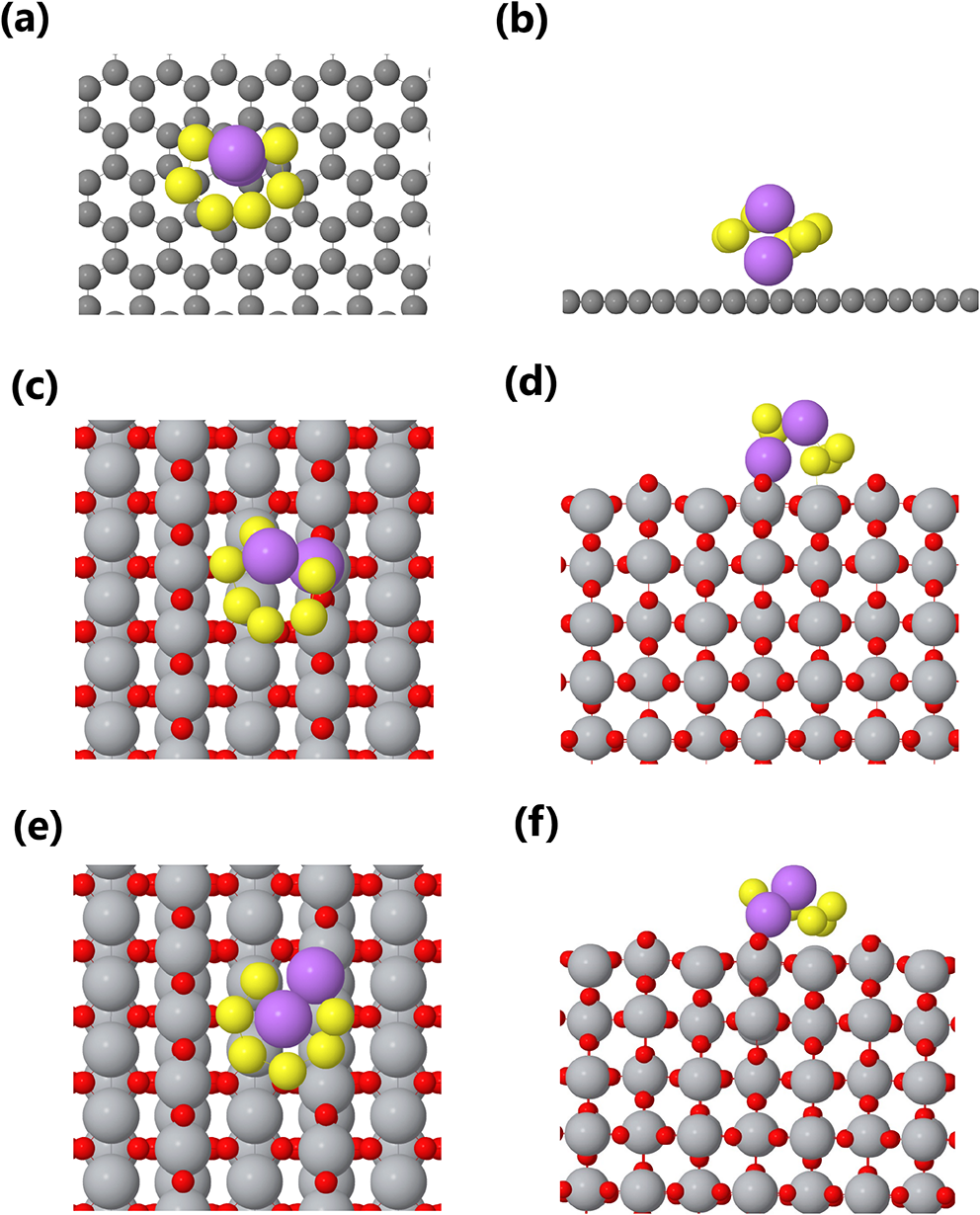
Top (left column) and side (right column) views of the relaxed configurations for lithium polysulfide adsorption on different surfaces. (a, b) Graphene, (c, d) rutile TiO_2_ (110), and (e, f) rutile H–TiO_2_ (110). Lithium atoms are shown in purple, and sulfur atoms in yellow.

The free adsorption energies calculated for the three surfaces are summarized in Table 1. As expected, the binding strength increases progressively from graphene to TiO_2_ and is the highest for H–TiO_2_. The negative values of the adsorption energies indicate that the interactions are spontaneous and thermodynamically favorable. More negative adsorption energies—such as those observed for H-TiO_2_, correspond to stronger interactions between the adsorbate and the host surface. Strongly negative values are typically associated with chemisorption, whereas less negative values suggest physisorption.^55^ The high adsorption energy of Li_2_S_6_ on H–TiO_2_ further confirms that oxygen vacancies significantly enhance the anchoring ability toward LiPSs. Consequently, the dual-binding sites with more favorable adsorption energies are expected to suppress the polysulfide shuttling effect effectively.

**Table 1:** Calculated Free Adsorption Energies at 300 K for Li_2_S_6_ Polysulfide Species on the Three Model Surfaces (Graphene, Rutile TiO_2_ (110), and H-TiO_2_ (110))

surface	Δ*G*_ads_ [eV]
graphene	–1.398
TiO_2_	–2.880
H-TiO_2_	–3.114

Overall, structural and chemical analyses confirm that the electrospun PVDF–MXene–TMO interlayers and the hydrogen-treated H-TiO_2_/S cathodes form a porous, defect-rich, and catalytically active framework. This architecture enables effective LiPS confinement, accelerates redox conversion, and preserves electrode integrity, thereby establishing a strong foundation for the enhanced electrochemical performance discussed in the following section.

### Electrochemical Performance: Dual Comparison

#### Cycling Stability

Cycling performances of cells with different interlayers for the H-TiO_2_/S and C/S cathodes are shown in Figure 8. For the H–TiO_2_/S cathode, the PV-MS interlayer delivers the most stable performance, exhibiting a low capacity decay rate of 0.38% per cycle and retaining 81% of its initial capacity (from 1131.4 to 879.4 mAh g^–1^) after 50 cycles while maintaining a Coulombic efficiency above 98% throughout. This improvement arises from the strong synergy between the sulfur host and the interlayer. The hollow architecture, together with hydrogen-induced oxygen vacancies, forms a vacancy-rich and porous framework that promotes controlled sulfur release and strong chemical anchoring of LiPSs. This vacancy-assisted confinement mitigates abrupt LiPS dissolution, reduces polarization, and preserves active-material connectivity during cycling, complementing the polysulfide adsorption and catalytic activity provided by the MXene–SnO_2_ domains.

**Figure 8:**
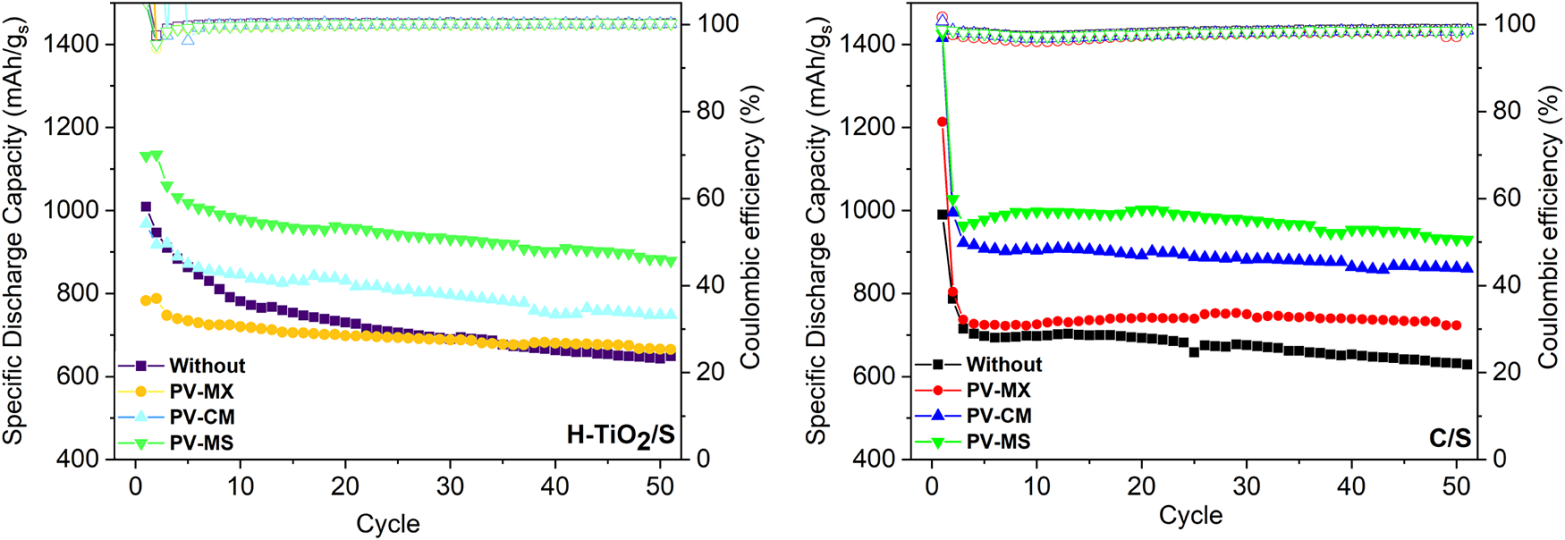
Cycling performance of cells with different interlayers over 50 cycles at 0.1 C for (a) the H–TiO_2_/S cathode and (b) the C/S cathode.

In contrast, cells employing PV-MX and PV-CM interlayers exhibit higher capacity decay rates (0.87 and 0.40% per cycle, respectively), indicating less effective shuttle suppression. The unexpectedly weaker long-term stability of the PV–CM interlayer—despite its high initial capacity (1328.7 mAh g^–1^)—is attributed to multiple coupled factors, including (i) restacking of MXene sheets leading to a loss of active sites, (ii) reduced interfacial contact with the cathode surface, and (iii) the presence of a suboptimal TiO_2_ phase within the MXene framework.^54,56^

A similar trend was observed for the conventional C/S cathode, where the PV-MS interlayer again outperformed the others, delivering the highest capacity retention (79%) and the lowest decay rate (0.41% per cycle), thereby ensuring stable capacity and high Coulombic efficiency throughout cycling. Meanwhile, the PV-MX and PV-CM interlayers exhibited faster degradation rates (0.81 and 0.78% per cycle, respectively) without achieving comparable stability. This comparative analysis highlights that the PV-MS interlayer provides the most effective suppression of polysulfide shuttling across both cathode systems, while the limited improvement observed for PV-CM underscores the importance of matching the interlayer chemistry with the cathode characteristics to achieve an optimal performance. The mechanistic differences between the interlayers become evident within the first 5–20 cycles, which is typically sufficient to diagnose shuttle-suppression efficiency, early-stage sulfur utilization, and stability trends in Li–S systems. In conventional Li–S cells, the initial 10–20 cycles represent the most critical region, where rapid capacity decay, severe polysulfide dissolution, and polarization buildup are most pronounced. In our study, the C/S cathode exhibits the expected sharp initial decline, whereas the PV-MS interlayer markedly suppresses this degradation, even at early cycles, demonstrating its ability to regulate LiPS redox reactions and stabilize electrode interfaces. Conversely, the H-TiO_2_/S displays a more gradual capacity evolution, consistent with controlled sulfur release from the hollow host. Therefore, the first 20–30 cycles already capture the intrinsic mechanistic differences between the interlayers, and the 50-cycle window is sufficient to reveal the dominant stability trends dictated by the interlayer chemistry and host–interlayer synergy.

The rate capability analysis (Figure 9) further highlights the synergistic benefits of integrating H–TiO_2_/S cathodes with multifunctional interlayers. Although the rate capability of the cells without interlayers suggests that the C/S cathode performs better than H–TiO_2_/S, the introduction of interlayers completely reverses this trend. Specifically, the H–TiO_2_/S cathode with the PV–MS interlayer delivers an initial capacity of 1004.8 mAh g^–1^ at 0.1 C and retains 855 mAh g^–1^ upon returning to 0.1 C. In contrast, the C/S cathode with the same interlayer starts at 967.3 mAh g^–1^ at 0.1 C but recovers only to 766.4 mAh g^–1^.

**Figure 9:**
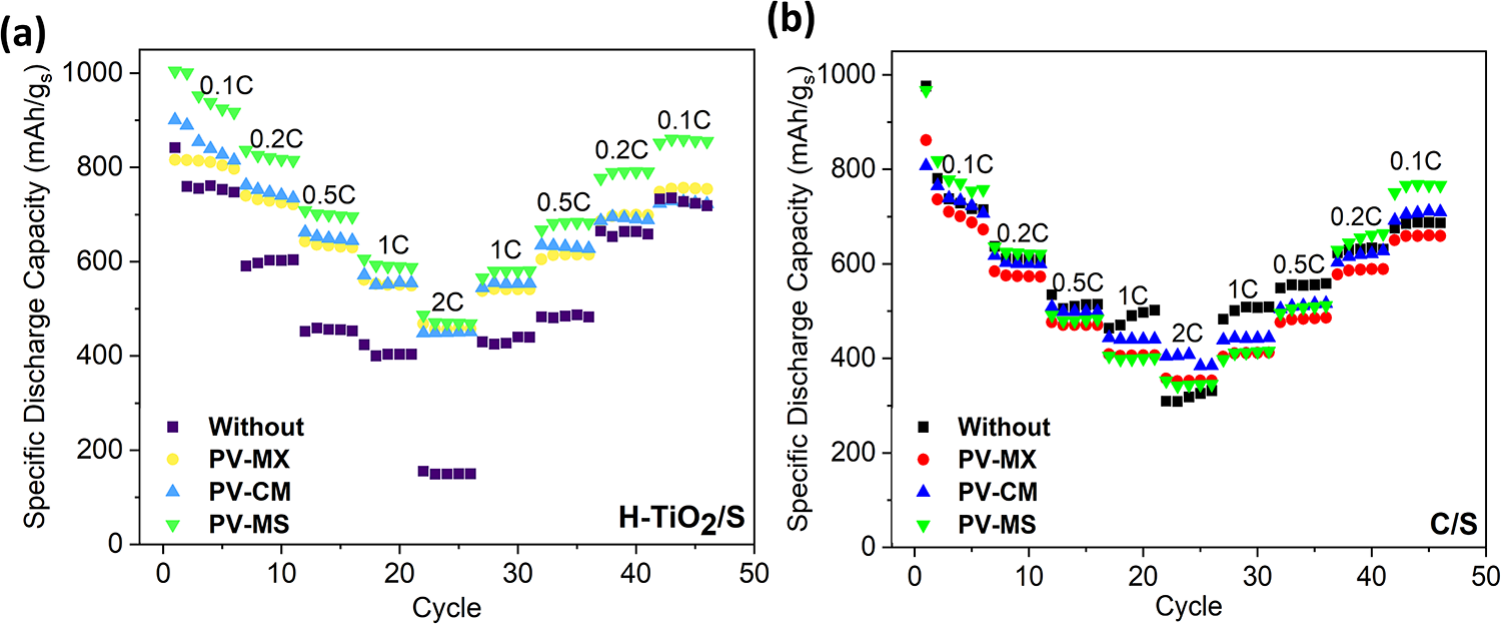
Rate capability of cells with different interlayers: (a) H-TiO_2_/S cathode and (b) C/S cathode.

This direct comparison clearly demonstrates that the hollow TiO_2_ framework exhibits a superior ability to sustain a high-rate operation, whereas the conventional C/S cathode suffers more pronounced capacity fading at elevated current densities. Even at 2 C, the H–TiO_2_/S cathode with PV–MS achieves 500 mAh g^–1^, while its C/S counterpart reaches only 350 mAh g^–1^.

The outstanding performance of H–TiO_2_ arises from its hollow nanostructure, which provides abundant active sites for polysulfide adsorption, shortens Li^+^ diffusion pathways, and enhances charge-transfer kinetics. When coupled with the conductive and catalytic properties of the PV–MS interlayer, this structural advantage yields markedly improved rate capability across all current densities. The H–TiO_2_/S electrode not only sustains stable capacity under demanding cycling conditions but also exhibits excellent capacity recovery, underscoring its mechanical and electrochemical durability.

The cyclic voltammetry (CV) analysis further highlights the synergistic benefits of integrating H–TiO_2_/S cathodes with multifunctional interlayers. During the cathodic scan, two distinct reduction peaks appear at approximately 2.3 V (corresponding to the reduction of S_8_ to higher-order polysulfides, Li_2_S_*x*_, 4 < *x* < 8) and around 2.0 V (associated with the formation of Li_2_S_2_ and Li_2_S). In the subsequent anodic scan, two oxidation peaks are observed at 2.45–2.55 V, corresponding to the conversion of Li_2_S_2_ and Li_2_S back to higher-order polysulfides and ultimately to sulfur.^57^ The reduction peak at 1.75 V and oxidation peak at 1.95 V correspond to Li^+^ insertion into the rutile TiO_2_ shell.^58^

The evolution of these polysulfides is also reflected in the charge/discharge profiles provided in Figures S2 and S3 of the Supporting Information.

As shown in Figure 10, among all configurations tested, the H–TiO_2_/S electrode equipped with the PV–MS interlayer exhibits the highest anodic and cathodic peak currents, accompanied by a noticeable leftward shift in the anodic peaks and a corresponding rightward shift in the cathodic peaks. The performance ranking follows the order: PV–MS > PV–CM > PV–MX, with the beneficial effect being more pronounced for the H–TiO_2_ host than for the conventional C/S cathode.

**Figure 10:**
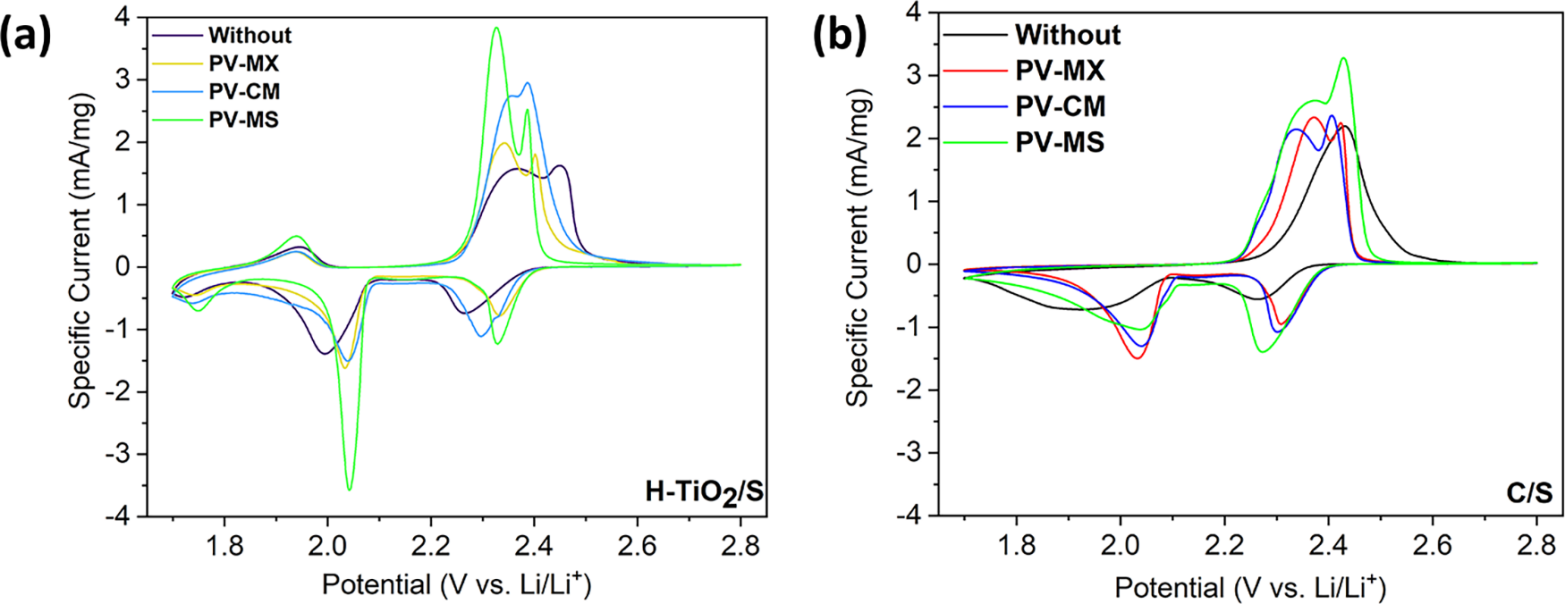
Cyclic voltammetry (CV) curves recorded at a scan rate of 0.1 mV s^–1^ in the voltage window of 1.8–2.6 V for cells incorporating different interlayers: (a) H–TiO_2_/S cathode and (b) C/S cathode.

The leftward shift of the anodic peaks indicates a reduced overpotential: hydrogen-induced oxygen vacancies and defects in TiO_2_ enhance its electronic conductivity and create additional active sites, thereby lowering the energy barrier for the oxidation of lithium sulfide species. The MXene/TMO interlayer further accelerates charge transfer by facilitating electron and ion transport and providing supplementary catalytic sites, collectively contributing to faster oxidation and promoting the observed shift.

The increased anodic and cathodic peak currents originate from a combination of (i) enlarged electroactive surface area—enabled by the hollow TiO_2_ architecture and the uniformly dispersed MXene/TMO network, and (ii) enhanced electrical conductivity, which together promote rapid electron movement. Meanwhile, the PV–MS interlayer effectively traps and catalyzes the conversion of lithium polysulfides, ensuring more complete redox processes and yielding higher current responses.

Together, these CV results clearly demonstrate that the integration of hollow-structured H–TiO_2_ with the PV–MS interlayer maximizes reaction kinetics and polysulfide regulation, providing a compelling mechanistic basis for its superior electrochemical performance in Li–S batteries.

CV at variable scan rates provides valuable insight into the transport properties of Li–S batteries, as the Randles–Sevcik equation enables extraction of the lithium-ion diffusion coefficient (*D*_Li^+^_) and allows differentiation between diffusion- and kinetics-limited processes. In this work, CV measurements were performed between 0.1 and 0.4 mV s^–1^, and the corresponding curves are shown in Figure 11. The relationship between the peak current (*I*_p_) and the square root of the scan rate (ν^1/2^) was used to determine the slope, which reflects the redox kinetics and diffusion-controlled character of the system. The governing equation is provided below.^59^

**Figure 11:**
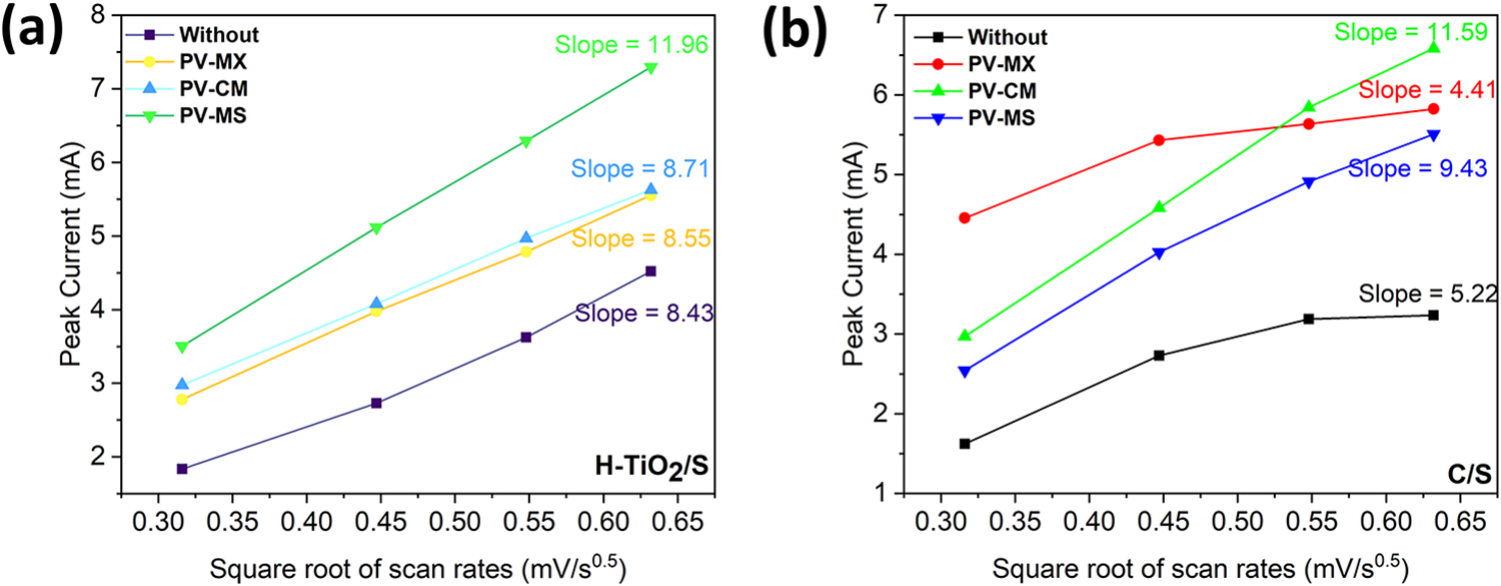
Cyclic voltammetry at different scan rates for cells with different interlayers: (a) H-TiO_2_/S cathode and (b) C/S cathode.

In Figure 11a, the slight upward trend in current with increasing scan rate indicates mixed charge-storage behavior, with both diffusion-controlled and capacitive (CPE) contributions.^60^ It also confirms that the reaction kinetics remain fast without reaching kinetic limitations even at higher scan rates. The H–TiO_2_/S cathode combined with the PV–MS interlayer exhibits the steepest slope, signifying superior charge-transfer kinetics compared to the bare C/S cathode and the other interlayer configurations. This behavior arises from the synergistic combination of H-TiO_2_ (which offers abundant active sites, vacancy-assisted ion diffusion, and enhanced electronic conductivity) and the PV–MS fibrous architecture, which provides efficient ion-transport pathways.

In contrast, the C/S cathode (Figure 11b) displays a downward trend in peak current with ν^1/2^, suggesting finite diffusion or the onset of kinetic limitations at higher scan rates (around 0.45 mV s^–1/2^). This indicates that the electrode struggles to sustain rapid charge transfer or ion transport at elevated scan rates.^61^

$$i_{p}=2.69\times 10^{5}n^{3/2}\mathrm{AC}\sqrt{\mathrm{Dv}}$$

where *i*_p_ is peak current, *n* is the number of electrons transferred, *A* is the electrode area, *C* is the Li^+^ concentration, *D* is the Li^+^ diffusion coefficient, and *v* is the scan rate. Using the Randles–Sevcik equation, diffusion coefficients were subsequently calculated (Table 2). The H–TiO_2_+PV–MS system delivered a diffusion coefficient of 1.126 × 10^–9^ cm^2^ s^–1^, nearly twice that of the cell without an interlayer, confirming a substantial enhancement in Li^+^ transport. In contrast, for the conventional C/S cathode, the PV–CM interlayer achieved the highest diffusion coefficient (1.058 × 10^–9^ cm^2^ s^–1^), outperforming the PV–MS counterpart.

**Table 2:** Calculated Diffusion Coefficients Derived from the Randles–Ševčík Equation for the H–TiO_2_/S and C/S Cathodes with Different Interlayers

interlayer	H-TiO_2_ cathode	C/S cathode
without	5.60 × 10^–10^	2.15 × 10^–10^
PV-MX	5.75 × 10^–10^	1.53 × 10^–10^
PV-CM	5.98 × 10^–10^	1.058 × 10^–9^
PV-MS	1.126 × 10^–9^	7.01 × 10^–10^

This comparison highlights that while PV–MS is more effective when paired with the H–TiO_2_/S cathode due to improved interfacial charge transfer, PV–CM proves more advantageous for the C/S cathode, where polysulfide shuttling is the dominant limitation. Taken together, these results demonstrate that carefully tailoring the interlayer–cathode combination is essential: the appropriate pairing not only maximizes ion diffusivity but also effectively addresses the distinct performance constraints of each cathode system.

To further evaluate lithium-ion transport in Li–S cells, the galvanostatic intermittent titration technique (GITT) was employed in addition to CV and Randles–Sevcik analysis. Unlike CV, which provides diffusion coefficients based on peak-current dependencies, GITT directly probes the chemical diffusion coefficient (*D*_Li^+^_) by applying controlled current pulses and analyzing the subsequent voltage relaxation response in accordance with Fick’s law.^62^ This approach enables a more accurate assessment of ion mobility across the electrode–electrolyte interface during electrochemical reactions.

Figure 12a,b presents the GITT profiles recorded during the initial discharge of Li–S batteries equipped with PV–MS interlayers paired with either H–TiO_2_/S or C/S cathodes, while Figure 12c,d shows the corresponding variation of the lithium-ion diffusion coefficient (*D*_Li^+^_) as a function of potential. In the H–TiO_2_/S system, the PV–MS interlayer delivers the highest diffusion coefficient values, indicating accelerated Li^+^ transport and reduced kinetic limitations.

**Figure 12:**
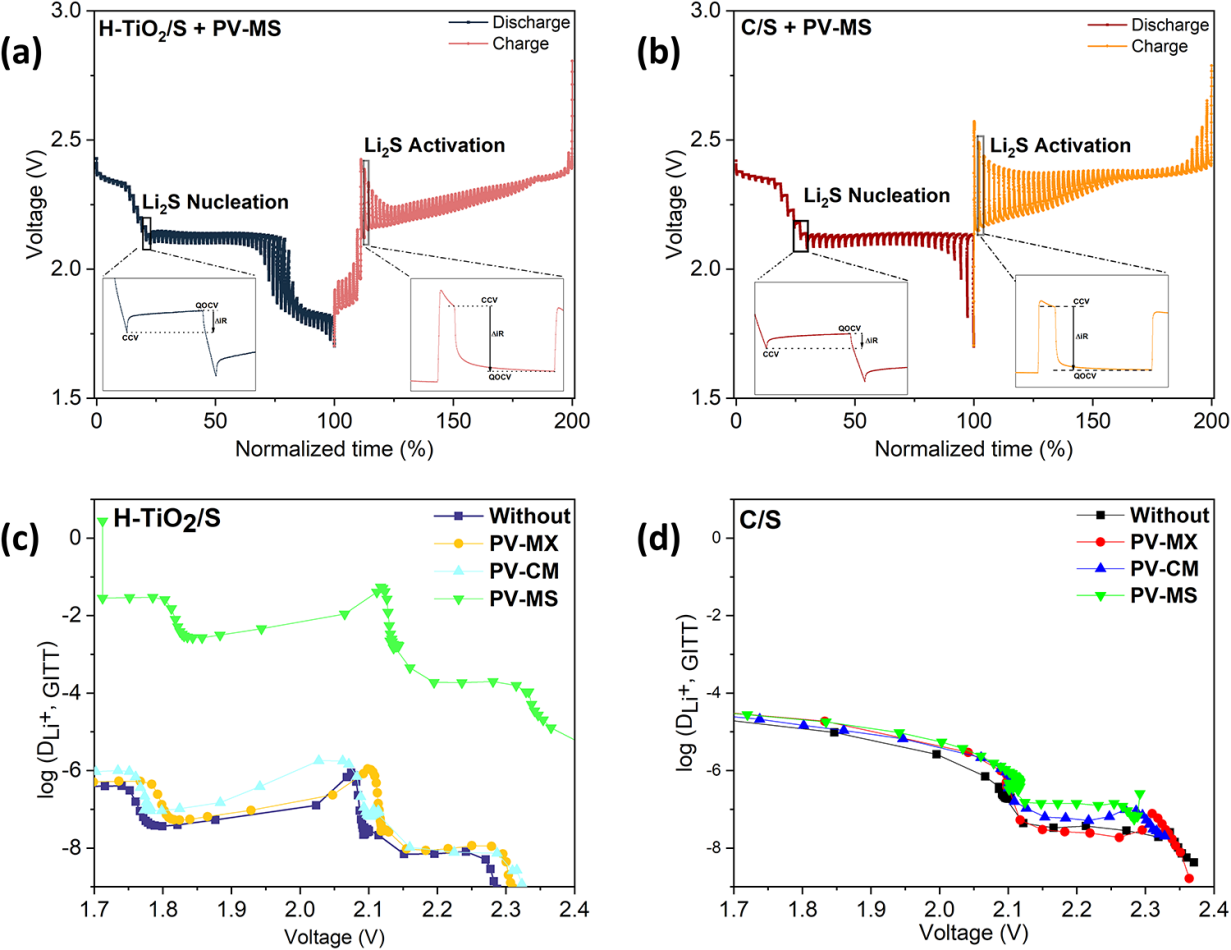
GITT profiles recorded during the initial discharge process of cells containing the PV–MS interlayer with (a) H–TiO_2_/S and (b) C/S cathodes. (c, d) Corresponding lithium-ion diffusion coefficients (*D*_Li^+^_) as a function of potential, extracted from the GITT measurements in (a, b).

Across both cathode types—and independent of the measurement technique—the diffusion coefficients follow a consistent trend of PV–MS > PV–CM > PV–MX > without interlayer, demonstrating that the interlayer chemistry plays a decisive role in enhancing ion mobility. The superior *D*_Li^+^_ observed for the H–TiO_2_/S–PV–MS configuration arises from the synergistic combination of strong polysulfide adsorption by the H-TiO_2_ host and the open, porous fibrous architecture of the PV–MS interlayer. Together, these features facilitate rapid ion diffusion, reduce polarization, and promote improved redox kinetics.^63^

EIS was used to deconvolute the interfacial resistances and catalytic behavior of the cells (Figure 13). The impedance response is modeled with R_1_ (electrolyte/contact resistance) in series with two midfrequency elements assigned to the lithium/electrolyte interfacial resistance (R_2_) and the polysulfide charge-transfer resistance (R_3_), followed by a low-frequency Warburg tail associated with Li^+^ diffusion.^64^ Incorporation of the PV–MS interlayer into H–TiO_2_/S cells produces a modest reduction in R_1_, implying improved ionic conduction and reduced interfacial polarization near the separator. However, the most striking change is observed in R_3_: the polysulfide charge-transfer resistance decreases from 4.5 Ω in the reference cell to 0.31 Ω with the PV–MS interlayer (93% reduction), signifying the near elimination of the kinetic barrier for LiPS redox conversion. For comparison, C/S cells with the same interlayer show a reduction of R_3_ from 8.4 to 2.9 Ω (65% reduction), underscoring that the H–TiO_2_ framework amplifies the catalytic benefit of the interlayer. Detailed resistance values are provided in Supporting Information, Table S1.

**Figure 13:**
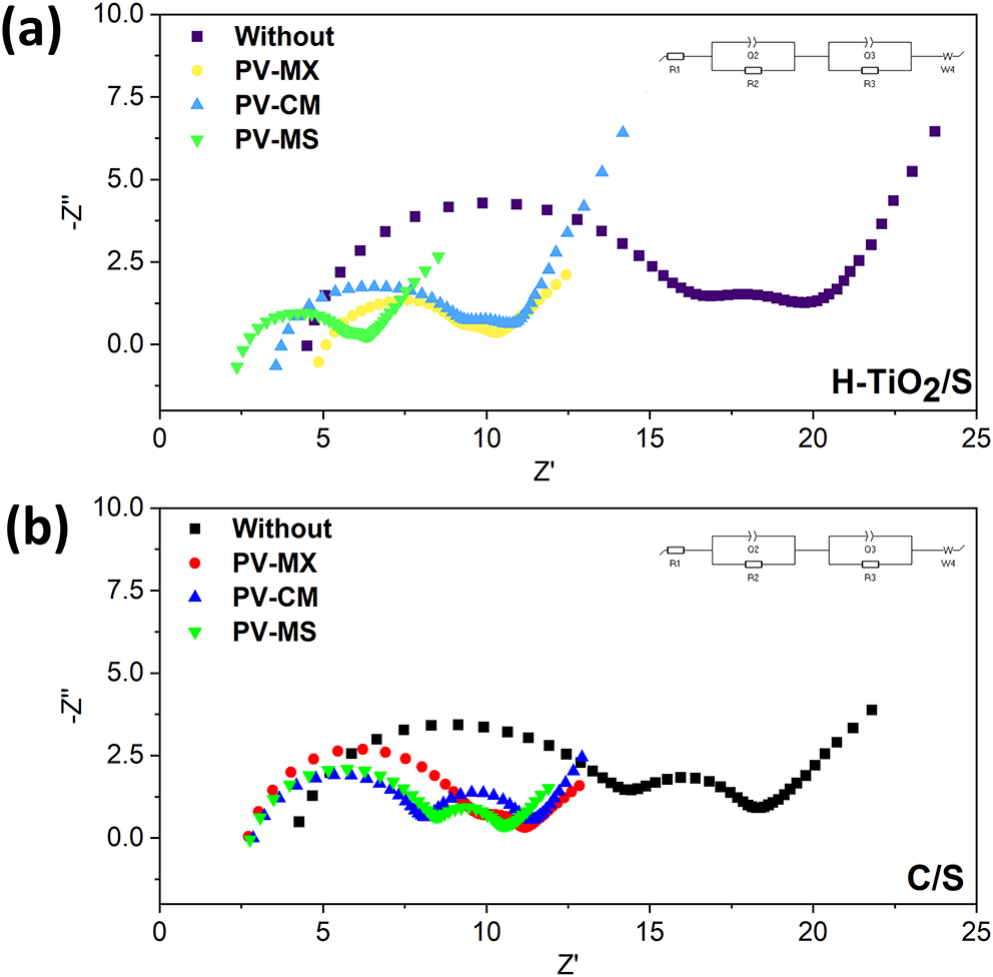
Electrochemical Impedance Spectroscopy (EIS) analysis of cells with different interlayers: (a) H-TiO_2_/S cathode and (b) C/S cathode.

Mechanistically, this dramatic improvement arises from a synergistic (dual) effect: (i) the H–TiO_2_ provides intrinsic catalytic sites—hydrogen-induced Ti^3+^ centers and oxygen vacancies—which lower the activation energy for polysulfide conversion and increase electronic/ionic accessibility of the active surface; and (ii) the PV–MS interlayer functions as a chemically active reservoir and physical scaffold that adsorbs soluble LiPSs, promotes three-dimensional nucleation/deposition of discharge products, and prevents rapid passivation of the cathode.^65^ The combined action accelerates interfacial charge transfer (reduced R_3_) and flattens the Warburg response (improved diffusion kinetics), while the large semicircles and increasing R_3_ in reference cells are consistent with insulating Li_2_S passivation and depletion of soluble species.^66^

The interaction between LiPS and the interlayers was systematically evaluated via adsorption experiments. Equal amounts of each interlayer were immersed in a 2 mM Li_2_S_6_ solution for 3 days, and the resulting chemical states of the solutions were compared with a control (without an interlayer). Visual inspection (Figure 14a–c) revealed pronounced color changes indicative of polysulfide removal: the PV–MS interlayer produced the most significant fading, rendering the solution nearly transparent, followed by PV–CM. MXene–TMO interlayers exhibited the largest observable color change, reflecting their strong adsorption capability. This enhanced performance arises from the abundant surface functional groups and expanded interlayer spacing of the MXene–TMO structure, which provide a high density of active sites for LiPS adsorption. Furthermore, the combination of MXene’s high electronic conductivity and the catalytic/adsorptive properties of TMO promotes rapid and efficient polysulfide capture, outperforming either component alone.^67^

**Figure 14:**
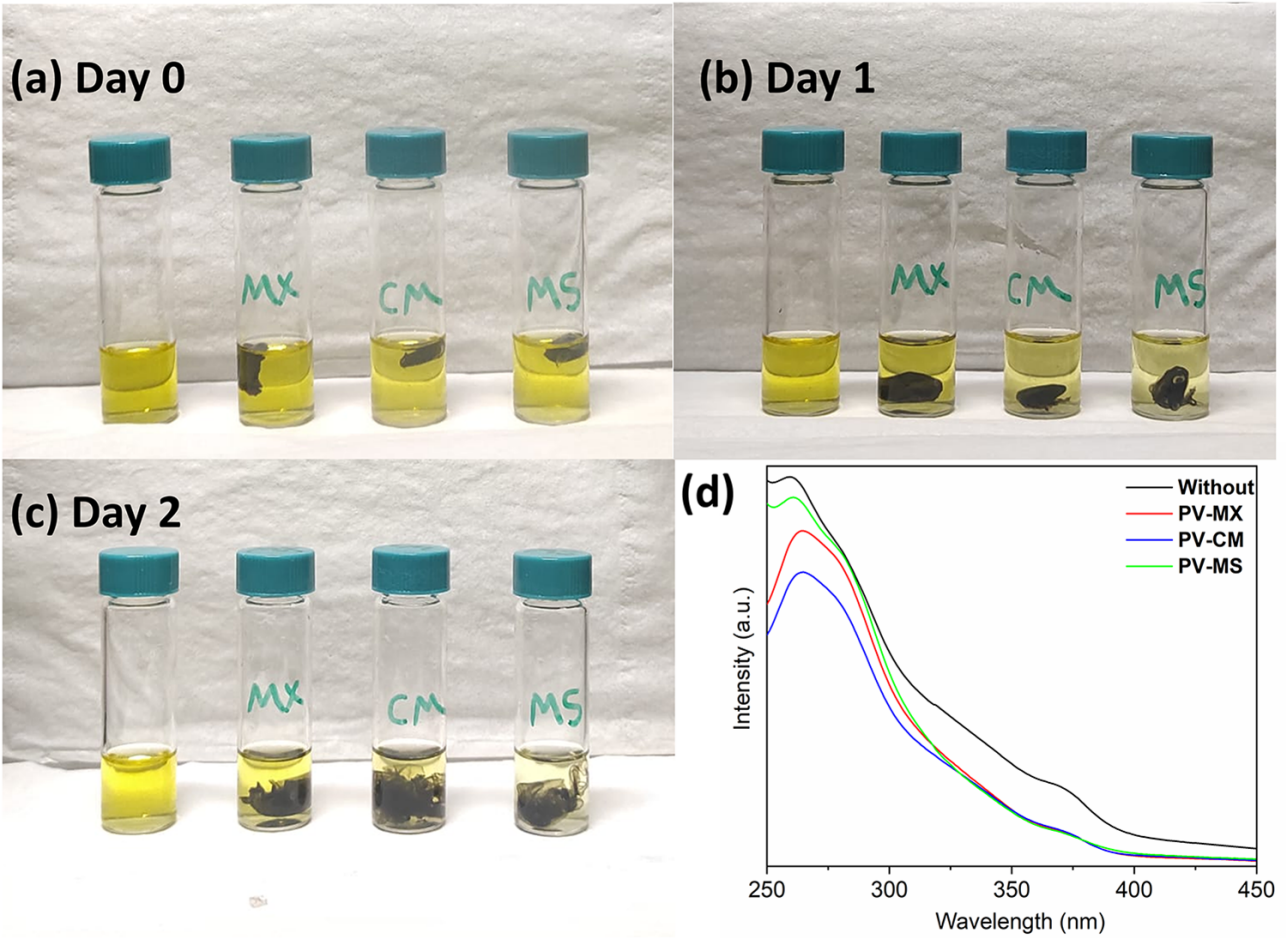
Polysulfide adsorption behavior of the interlayers over 3 days: (a) freshly prepared Li_2_S_6_ solution (Day 0), (b) after 1 day, (c) after 2 days, and (d) UV–VIS absorption spectra of the Li_2_S_6_ solution after 3 days of adsorption.

UV–VIS absorption spectra (Figure 14d) corroborate these observations: the characteristic peak of S_6_^–2^ at 260 nm decreases markedly upon interlayer addition, confirming strong interactions between Li_2_S_6_ and the interlayers. Interestingly, despite the most pronounced color fading with PV–MS, its absorption peak was not the lowest. This apparent discrepancy can be attributed to the formation of surface-bound polysulfide intermediates or complexes on MXene–SnO_2_, which exhibit distinct absorption features that can exceed those of free Li_2_S_6_.^68^ This effect implies that while the visual color change reflects efficient polysulfide removal, UV–VIS absorbance alone may overestimate the residual free polysulfide concentration.

The comprehensive electrochemical analysis reveals that the H-TiO_2_/S cathode intrinsically enables controlled polysulfide release and accelerated redox kinetics through the presence of Ti^3+^ centers and abundant oxygen vacancies, thereby minimizing the number of sluggish conversion steps. Complementarily, the PV–MS interlayer plays a decisive role in suppressing polysulfide shuttling and enhancing electronic/ionic conductivity via its conductive MXene framework and polar TMO sites. When integrated, these two design strategies act cooperatively, yielding a synergistic improvement in interfacial stability and long-term cycling performance that neither component could achieve alone. To the best of our knowledge, this is the first report demonstrating a dual-engineered Li–S cathode system in which H–TiO_2_ and a functional interlayer operate in tandem to simultaneously accelerate LiPS conversion, regulate their spatial distribution, and suppress parasitic shuttling, thereby establishing a new paradigm for cathode-interlayer codesign in advanced Li–S batteries.

## Conclusion

In this work, we introduce a dual-engineered Li–S battery architecture comprising an H-TiO_2_/S host and a conductive, catalytic PV–MS interlayer to overcome the long-standing polysulfide shuttle and sluggish conversion kinetics in Li–S batteries. DFT calculations further substantiate this effect, revealing adsorption energy trends of |Δ*G*_ads_|_H–TiO_2__ > |Δ*G*_ads_|_TiO_2__ > |Δ*G*_ads_|_graphene_, indicating that H-TiO_2_ exhibits the strongest LiPS adsorption affinity through dual-site binding of sulfur and lithium atoms. The H-TiO_2_ framework, enriched in Ti^3+^/oxygen vacancies, provides enhanced conductivity and abundant polar sites for polysulfide adsorption, while the hollow architecture regulates sulfur confinement and promotes gradual Li_2_S deposition. Complementarily, the PV–MS interlayer suppresses shuttle migration, facilitates charge transfer, and stabilizes the electrode/electrolyte interface.

The synergy between the host and interlayer yields performance metrics that far surpass those of conventional designs: Li^+^ diffusion coefficients nearly double, charge-transfer resistance decreases by 93% (4.5 → 0.31 Ω), long-term cycling stability improves markedly (capacity retention >80% after extended cycling, compared to 64% in interlayer-free baselines), and rate capability is significantly enhanced due to faster ion/electron transport. These findings establish the H-TiO_2_–PV–MS system as a benchmark design for high-performance Li–S batteries and offer a broadly applicable blueprint: defect-engineered sulfur hosts coupled with multifunctional conductive–catalytic interlayers can decisively mitigate the shuttle effect and unlock durable, fast, and energy-dense Li–S chemistries.

## Experimental Section

### Materials

In order to prepare the C/S cathode material, sublimed sulfur (S_8_) was obtained from Sigma-Aldrich, and Super P was obtained from Imerys. The LA132 binder was purchased from Chengdu Yindile Power Source Science and Technology Co., Ltd.

The H-TiO_2_ cathode material was prepared using ECP600JD Ketjen Black from AkzoNobel, Pim-L acetylene black from IRPC, and Super P carbon black from TIMCAL. Polyvinylidene fluoride (PVDF) was supplied by MTI Corporation (Batch No.: 130507), and *N*-methyl-2-pyrrolidone (NMP) was purchased from Sigma-Aldrich (M79603-1L, Lot No. SZBD1920 V, CAS: 872-50-4).

For the electrolyte, bis­(trifluoromethane)­sulfonimide lithium salt (LiTFSI, 99.95% trace metals basis), LiNO_3_ (99.99% trace metals basis), 1,2-dimethoxyethane (DME, anhydrous, 99.5%, inhibitor-free), and 1,3-dioxolane (DOL, anhydrous, 99.8%, containing 75 ppm of BHT as inhibitor) were purchased from Sigma-Aldrich.

For the interlayers, PVDF (*M*_w_ = 380 000 g/mol) powder was purchased from Solvay. Tetrahydrofuran (THF), *N*,*N*-dimethylacetamide (DMAc, 99%), *N*,*N*-dimethylformamide (DMF, 99%), and acetone were also purchased from Sigma-Aldrich.

### Synthesis of Hollow-Structured H-TiO_2_

Hollow-structured H–TiO_2_ was prepared through a hard-templating method that involved the synthesis and subsequent etching of SiO_2_ templates. The overall process included four main steps: (i) preparation of SiO_2_ spheres, (ii) coating with TiO_2_ to form SiO_2_@TiO_2_ core–shell structures, (iii) removal of the SiO_2_ cores to obtain hollow TiO_2_ spheres, and (iv) hydrogen treatment to form conductive H–TiO_2_.

#### Preparation of SiO_2_ Spheres

Monodisperse SiO_2_ spheres were synthesized via a modified Stöber method, following Li et al.^69^ Briefly, tetraethyl orthosilicate (TEOS) was hydrolyzed in a mixture of ethanol, deionized water, and ammonia solution under continuous stirring at room temperature for 24 h. The resulting SiO_2_ particles were collected by centrifugation, thoroughly washed with ethanol and water, and dried at 110 °C overnight.

#### Synthesis of SiO_2_@TiO_2_ Core–Shell Structures

The as-prepared SiO_2_ spheres (0.25 g) were dispersed in ethanol and ultrasonically sonicated to ensure a uniform suspension. Ammonia and titanium butoxide (TBOT) were then added as the Ti precursor, and the mixture was stirred for 24 h in a 45 °C water bath. The resulting core–shell particles were collected by centrifugation, washed, dried at 110 °C, and subsequently calcined in air at 700 °C for 2 h to crystallize the TiO_2_ shell.

#### Synthesis of Hollow-Structured TiO_2_ Spheres

The SiO_2_ cores were selectively removed by chemical etching. The SiO_2_@TiO_2_ powder was treated with a 1 M NaOH solution at 90 °C for 3 h under continuous stirring. The etched product was repeatedly washed with deionized water until a neutral pH was reached and then dried at 110 °C overnight.

#### Synthesis of Hollow-Structured H-TiO_2_

To enhance the electrical conductivity, hollow TiO_2_ was annealed at 500 °C for 3 h in a 5% H_2_/Ar atmosphere at a flow rate of 80 mL min^–1^, yielding the final H–TiO_2_ with enriched Ti^3+^ and oxygen vacancies.

### Synthesis of Hollow-Structured H-TiO_2_/S Cathode

Composite electrodes for electrochemical studies were prepared by doctor-blade coating a slurry onto carbon-coated aluminum foil current collectors. The synthesized TiO_2_–S composite served as the active cathode material, while ECP600JD Ketjen black, Pim-L acetylene black, and Super P carbon black were used as the conductive agents. PVDF was dissolved in NMP and used as the binder.

The slurry composition is as follows: 80 wt %, ECP600JD Ketjen black 2 wt %, Pim-L acetylene black 8 wt %, and polyvinylidene fluoride 10 W %. The total solid/NMP ratio is 1:3. The resulting electrode films had a thickness of 40 μm, and the active sulfur loading was 1.5–2.0 mg cm^2^.

The TMO/S composite cathode slurry was mixed using a planetary centrifugal vacuum mixer (ARV-310, Thinky) at 600 and 900 rpm for 10 min, followed by 15 min ultrasonication, and then mixed again at 900 rpm for 10 min to achieve complete homogenization. The homogeneous slurry was then coated onto thin aluminum foil current collectors.

The coated films were dried at 35 °C in a vacuum oven (VO-300, As One Corporation) for 10–12 h, followed by mechanical pressing using a roller press (MSK-HRP-MR100A, MTI Corporation) to improve particle–particle and particle–collector contact.

Finally, the electrode films were punched into 13 mm diameter disks for assembly into coin cells.

### Synthesis of MXene and MXene-TMO Particles

MXene synthesis was conducted by following a minimally intensive layer delamination (MILD) method. The precursor Ti_3_AlC_2_ MAX phase (Laizhou Kai Kai Ceramic Materials Co., Ltd.), a hexagonal carbide/nitride with the general formula M_n+1_AX_n_, was selectively etched to obtain Ti_3_C_2_T_*x*_ MXene. In a standard procedure, 3.2 g of lithium fluoride (LiF, Sigma-Aldrich) was dissolved in 40 mL of 9 M hydrochloric acid (HCl, 37%, VWR) to form the etching solution, into which 2 g of MAX phase powder was gradually added under vigorous stirring. The etching process was carried out at 50 °C for 48 h. After completion, the reaction mixture was centrifuged at 1500 rcf for 5 min, the supernatant was discarded, and the sediment was repeatedly washed with 40 mL of ultrapure water. The washing–centrifugation cycle was performed five times until the supernatant reached a pH of 6.

The resulting suspension was homogenized by using a vortex mixer for 30 min, followed by ultrasonication for 1 h in an ice bath under continuous argon (Ar) bubbling to promote delamination into few- and single-layer nanosheets. Subsequently, the dispersion was centrifuged at 1500 rcf for 30 min to collect the exfoliated MXene nanosheets in the supernatant. A final centrifugation step at 15,000 rcf for 30 min was performed to isolate the nanosheets.

For calcination, the MXene powders were heated to 350 °C at a rate of 15 °C/min and maintained for 2 h. Post-treatment characterization by SEM and XRD confirmed the formation of TiO_2_ nanoparticles on the surface and between MXene flakes. Detailed characterization results are provided in the Supporting Information.

MXene–SnO_2_ nanoparticles were synthesized by dissolving SnCl_4_·5H_2_O (180 mg) in 20 mL of water, adjusting the pH to 8 with NH_3_·H_2_, and then slowly adding the solution into 80 mL of the Ti_3_C_2_T_*x*_ MXene colloid (1 mg/mL) under vigorous stirring and ultrasonication in an ice bath for 1 h. The mixture was subsequently hydrothermally treated at 120 °C for 6 h, and the resulting product was collected by centrifugation and washed three times. The product was then diluted, frozen, freeze-dried into powder, and stored at low temperature.

### Characterization

Sample morphologies were investigated using a FEI Quanta 650 field-emission SEM instrument (Nano SEM 230). Surface topography was primarily observed using the secondary electron (ETD) detector, while compositional contrast was obtained via the concentric backscattering (CBS) mode. Elemental mapping and EDS analysis were performed on a ZEISS GeminiSEM 460. The surface chemical states were probed by X-ray photoelectron spectroscopy (XPS, PHI Quantum 2000), in which Ar sputtering was applied for surface cleaning. Phase identification and structural analysis were carried out by using X-ray diffraction (XRD, Bruker D8). UV–VIS absorption spectra were recorded on a PerkinElmer Lambda 950 spectrophotometer. The surface morphology of the interlayers was examined by using a Zeiss LEO Supra 35VP SEM-FEG field-emission microscope operated at 3–5 kV.

### Computational DFT Analysis

The Gibbs free energy of adsorption for carbon, H-TiO_2_, and TiO_2_ was estimated as^70^

1 $$\Delta G_{\mathrm{ads}}\left(T\right)=\Delta E_{\mathrm{DFT}}+\Delta E_{\mathrm{zpe}}+\Delta F_{\mathrm{vib}}\left(T\right)$$

where Δ*E*_DFT_ is the adsorption energy as calculated by DFT at 0 K

2 $$\Delta E_{\mathrm{DFT}}=E_{\mathrm{ads}}-E_{\mathrm{surf}}-E_{\mathrm{gas}}$$

in which *E*_ads_ is the energy of the whole system composed of the polysulfide adsorbed on the surface, *E*_surf_ is the energy of the surface alone, and *E*_gas_ is the energy of the polysulfide molecule in the gas phase. The second and third terms in eq 1 include the effects of vibrations. In particular, the second term includes the difference of zero point energies, and was calculated assuming that the vibrational spectrum of the surface does not change upon adsorption of the polysulfide molecule, that is

3 $$\Delta E_{\mathrm{zpe}}=\sum_{\nu_{\mathrm{ads}}\;}\frac{h\nu_{\mathrm{ads}}}{2}-\sum_{\nu_{\mathrm{gas}}\;}\frac{h\nu_{\mathrm{gas}}}{2}$$

where ν_ads_ are the frequencies of the polysulfide only in the adsorbed configuration, ν_gas_ are those of the same molecule in the gas phase and *h* is the Planck constant. The same approximation was used to calculate the last term of eq 1, that is, the temperature-dependent vibrational contribution

4 $$\Delta F_{\mathrm{vib}}\left(T\right)=\sum_{\nu_{\mathrm{ads}}\;}\ln\left(1-e^{-h\nu_{\mathrm{ads}}/k_{B}T}\right)-\sum_{\nu_{\mathrm{gas}}\;}\ln\left(1-e^{-h\nu_{\mathrm{gas}}/k_{B}T}\right)$$

being *T* the temperature and *k*_B_ the Boltzmann constant.

Since our goal was to compare the differences of the results of eq 1 for the same Li_2_S_6_ polysulfide on three different surfaces, we neglected the translational and rotational contribution to the entropy of the molecule in the gas phase, as these contributions would just add the same constant to the three calculated values of Δ*G*_ads_(*T*). The DFT calculations were performed with the CP2K suite of codes.^71^ First, geometry optimization was performed for the three surfaces. We used a 10 × 10 repetition of the unit cell in the *xy* plane. For TiO_2_ we considered a total of 6 titanium layers and fixed the first three starting from the bottom. For both the TiO_2_ and graphene models, a vacuum of at least 20 Å between periodic images in the *z* direction in the supercell was used. Then, a geometry relaxation was done for the system with the polysulfide molecule adsorbed on the three surfaces. The structures were relaxed until all forces were below 10^–5^ Hartree/bohr and the maximum displacement was below 10^–3^ bohr. All calculations were performed at the γ point. To calculate the vibrational frequencies of the adsorbed polysulfide, we fixed all atoms of the substrate in the spirit of the approximation described above. For vibrational mode calculations, more refined geometry optimizations were performed by relaxing structures until all forces dropped below 10^–7^ Hartree/bohr and the maximum displacement was below 10^–5^ bohr.

We used the *r* ^2^SCAN exchange-correlation functional^72^ with rVV10 nonlocal correlation correction. The interactions between core and valence were described by the norm-conserving Goedecker–Teter–Hutter (GTH) pseudopotentials^73^ optimized for SCAN. The wave functions were expanded in double-ζ valence MOLOPT basis sets with one set of polarization functions for carbon and titanium and triple-ζ valence basis sets with two sets of polarization functions for oxygen, lithium, and sulfur.^74^ The scf calculations were performed with the orbital transform method implemented in CP2K, together with the conjugate gradient. A convergence threshold EPS_SCF of 10^–7^ was used in all cases with a plane wave energy cutoff of 800 Ry for the electronic density. For all calculations, the counterpoise correction by Boys and Bernardi^75^ was applied to account for basis set superposition errors.

### Electrospinning

The fabrication of the PVDF-MXene/TMO electrospinning ink consisted of two main stages. In the first stage, MXene/TMO powders were dispersed in a mixed solvent of DMF and acetone at a 7:3 ratio and ultrasonicated for 30 min to achieve homogeneous distribution. In parallel, PVDF was dissolved in a DMAC/DMF mixture (7:3, w/w) until a clear polymer solution was obtained. The MXene/TMO dispersion was then blended into the polymer solution, and the mixture was stirred overnight to ensure complete integration. The final ink composition contained an 80/20 weight ratio of polymer to MXene/TMO. Electrospinning was performed under 40% RH, with a voltage of 13 kV, a solution feed rate of 1.2 mL h^–1^, and a needle-to-collector distance of 15 cm. The produced interlayers showed an approximate thickness of ∼20 μm and an areal loading of ∼0.3 mg cm^2^, followed by drying at 60 °C for 3 h.

### Cell Assembly and Electrochemical Characterization

The C/S composite was prepared by mixing Super P and sulfur (60 wt % S) and heating the mixture at 155 °C for 12 h. The electrodes were fabricated by dispersing the composite with an LA132 binder (aqueous acrylonitrile copolymer latex) solution to form a slurry (C/S:binder = 9:1, w/w), casting it onto Al foil, air-drying, and then further drying at 60 °C for 12 h. Disks (Ø 13 mm) were punched and stored prior to cell assembly.

The electrolyte consisted of a 1:1 (v/v) mixture of DOL/DME containing 1 M LiTFSI and 1 wt % LiNO_3_ as an additive. The electrolyte-to-sulfur ratio (E/S) was kept constant at 20 ul/mgs.

The battery assembly route is shown in Figure 15. A free-standing electrospun PVDF/MXene–TMO membrane was used as the interlayer. It was laminated onto the separator on the cathode-facing side only, meaning that the interlayer was positioned on top of the separator and in direct contact with the cathode, while remaining fully isolated from the Li metal anode. Thus, during coin cell assembly, the stacking order was: Li metal anode/separator/interlayer/cathode.

**Figure 15:**
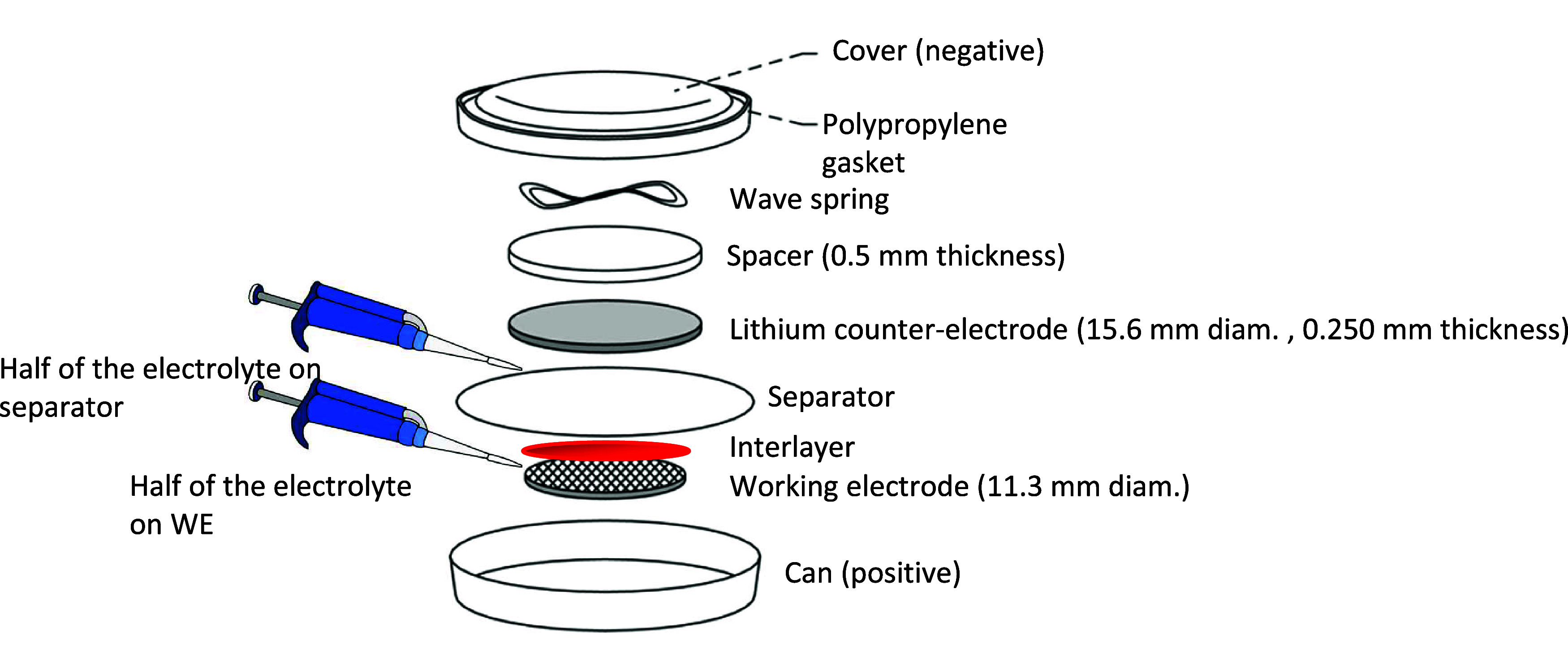
A schematic representation of a typical Li–S battery involving an interlayer.

Galvanostatic charge–discharge cycling and rate performance tests were carried out on a LAND battery testing system (CT-2001A, Wuhan Rambo Testing Equipment Co., Ltd.) over a potential window of 1.7–2.8 V. Prior to cycling, the cells were activated by one discharge at 0.02 C and a subsequent charge at 0.04 C. GITT experiments were performed on the same instrument with a current pulse of 0.1 C for 5 min, followed by a relaxation time of 30 min. Cyclic voltammetry (CV) and potentiostatic electrochemical impedance spectroscopy (PEIS) were conducted by using a VSP potentiostat (Bio-Logic, France). CV tests were carried out within a potential window of 1.7 to 2.8 V at a scan rate of 0.1 mV/s, with an amplitude of 10 V. The slopes used for calculating Li^+^ diffusion rates via the Randles–Sevcik equation were obtained from 0.1–0.4 mV/s scan rates. For EIS measurements, the applied perturbation was 10 mV, and data were collected in the frequency range of 100 kHz to 100 mHz at open-circuit potential. The chemical composition of the fiber surfaces was probed using an X-ray Photoelectron Spectrometer (XPS) system (Thermo Scientific K-α, monochromatic Al Kα source (*h*ν = 1486.61 eV)) over a binding energy range of 1350–0 eV.

For adsorption tests, a 2 mM Li_2_S_6_ solution was prepared by mixing Li_2_S and sulfur powder in a 1:5 molar ratio and dissolving the mixture in DOL. Thirty milligrams of each interlayer were immersed in the prepared solution and left to stand for 3 days. All procedures were conducted inside an argon-filled glovebox.

## Supplementary Material



## Notes

(a) XRD pattern and (b) SEM image of calcined MXene particles (Figure S1); charge/discharge curves for H-TiO_2_/S cathode comparison of without, PV-MX, PV-CM, and PV-MS interlayers at (a) first cycle, (b) fifth cycle, (c) 10th cycle, (d) 25th cycle, and (e) 50th cycle (Figure S2); charge/discharge curves for C/S cathode comparison of without, PV-MX, PV-CM, and PV-MS interlayers at (a) first cycle, (b) fifth cycle, (c) 10th cycle, (d) 25th cycle, and (e) 50th cycle (Figure S3); and resistance values of cells H-TiO_2_/S and C/S electrode–interlayer configuration (Table S1) (PDF)

## #

SCG, Bangkok 10800, Thailand
