# Supplementary material for: Enhanced Performance of Li–S Batteries via Dual Cathode–Interlayer Engineering: Hollow TiO2–Sulfur with Electrospun MXene–TMO Interlayers
Source: ACS Omega. 2026 Feb 17;11(8):13496–512. doi: 10.1021/acsomega.5c11112 (PMC12961462; doi:10.1021/acsomega.5c11112)
Supplement: Supplementary file 1 [file ao5c11112_si_001.pdf]

Enhanced Performance of Li–S Batteries via Dual Cathode–Interlayer Engineering: Hollow TiO_2 Sulfur with Electrospun MXene–TMO Interlayers

Busra Cetiner,[†] Shungui Deng,[‡] Cesare Roncaglia,[¶] Thanya Phraewphiphat, [#,§] Panpanat Tesatchabut,[§] Adisak Promwicha,[§] Daniele Passerone,[¶] Pimpa Limthongkul,[§] Jakob Heier,[‡] Begum Yarar Kaplan,[‖] Selmiye Alkan Gursel,[‖,†] and Alp Yurum[*,‖,†]

†Faculty of Engineering and Natural Sciences, Department of Materials Science and Nanoengineering, Sabancı University, 34956, Istanbul, Turkey

‡Laboratory for Functional Polymers, Empa, Swiss Federal Laboratories for Materials Science and Technology, Dubendorf, 8600, Switzerland

¶nanotech@surface, Empa, Swiss Federal Laboratories for Materials Science and Technology, Dubendorf, 8600, Switzerland

§National Energy Technology Center, National Science and Technology Development Agency, Pathumthani, 12120, Thailand

‖SUNUM Nanotechnology Research Centre, Sabancı University, 34956, Istanbul, Turkey

E-mail: alp.yurum@sabanciuniv.edu

[#] Present address: SCG, Bangkok, 10800, Thailand. E-mail: thanya.phraew@gmail.com

(a) 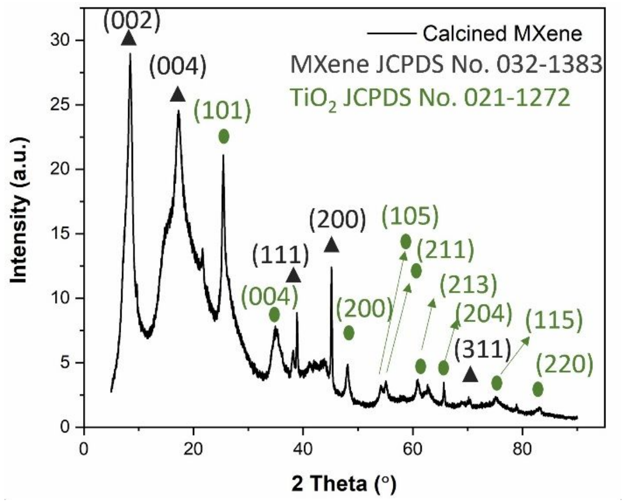

(b) 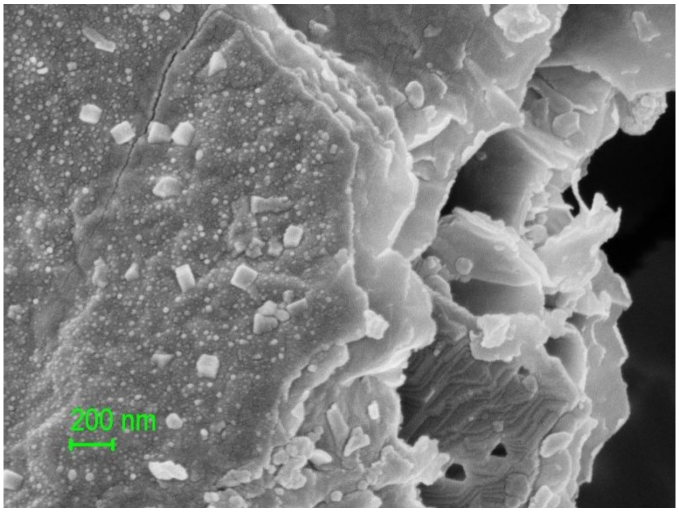

Figure S1. (a) XRD pattern, and (b) SEM image of Calcined MXene particles

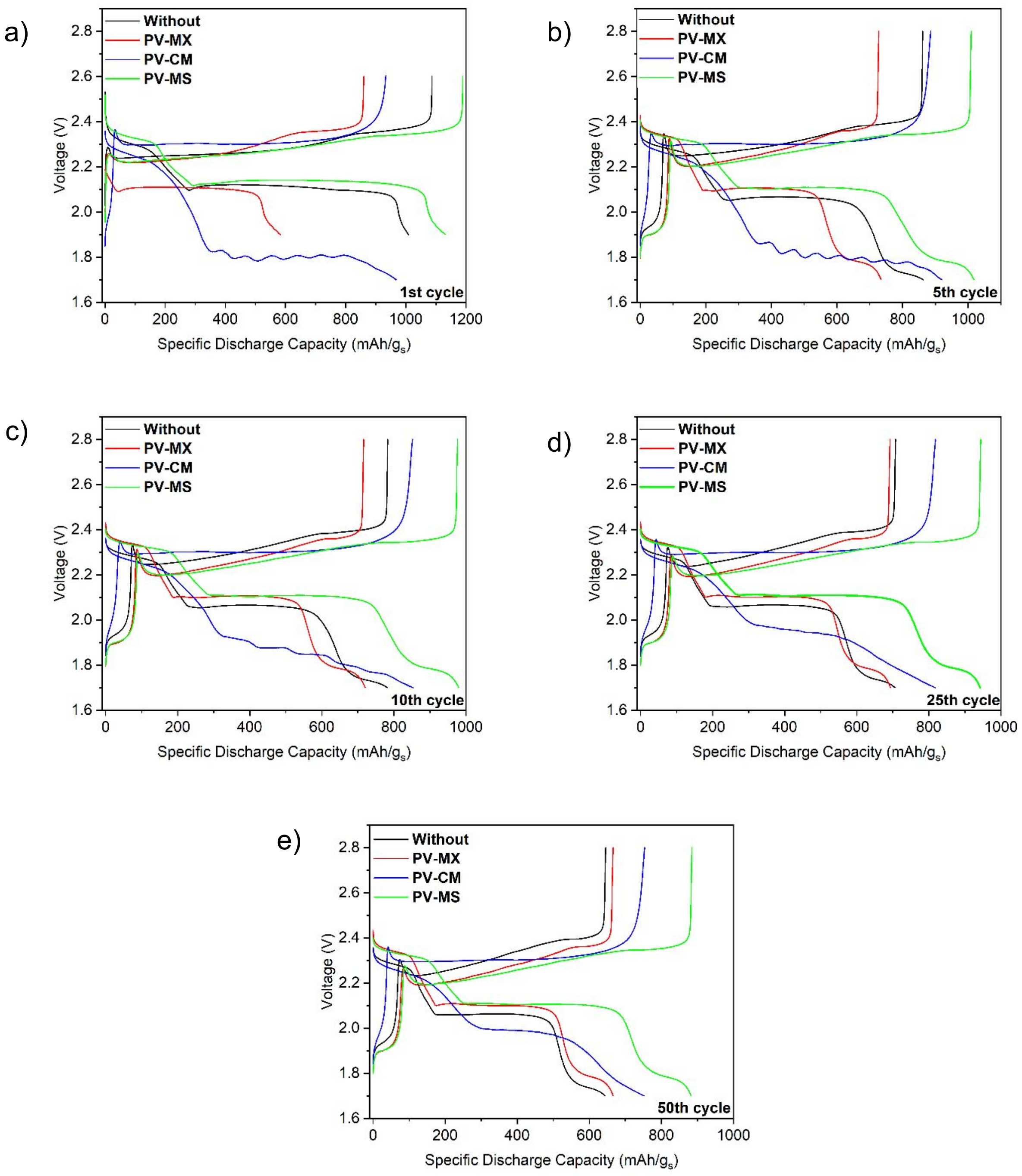

Figure S2. Charge/discharge curves for H-TiO_2/S cathode comparison of without, PV-MX, PV-CM, and PV-MS interlayers at a)1st cycle, b)5th cycle, c)10th cycle, d)25th cycle, and e)50th cycle.

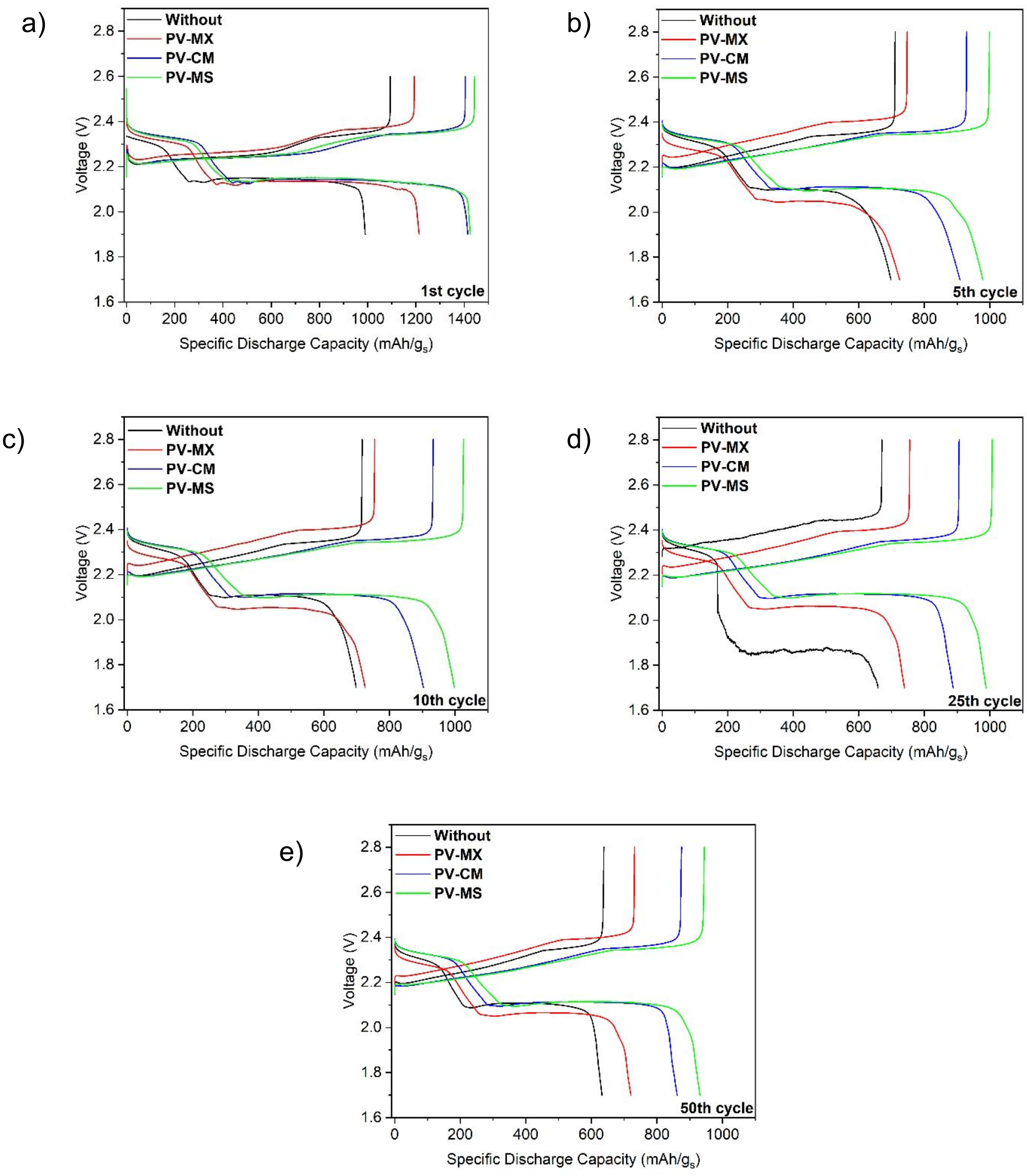

Figure S3. Charge/discharge curves for C/S cathode comparison of without, PV-MX, PV-CM, and PV-MS interlayers at a)1st cycle, b)5th cycle, c)10th cycle, d)25th cycle, and e)50th cycle.

Table S1. Resistance values of cells H-TiO_2/S and C/S electrode–interlayer configuration

	**H-TiO_2/S cathode**			**C/S cathode**		
**Cell Type**	**R1**	**R2**	**R3**	**R1**	**R2**	**R3**
Without	4.8	10	4.5	4.5	5.5	8.4
PV-MX	5.3	3.7	1.6	4	2.5	6
PV-CM	3.9	5.15	1.2	3.3	4.9	5.1
PV-MS	2.7	3.1	0.31	3.1	2.2	2.9
